# Burden of SARS-CoV-2 infection in healthcare workers during second wave in England and impact of vaccines: prospective multicentre cohort study (SIREN) and mathematical model

**DOI:** 10.1136/bmj-2022-070379

**Published:** 2022-07-20

**Authors:** Diane Pople, Edward J M Monk, Stephanie Evans, Sarah Foulkes, Jasmin Islam, Edgar Wellington, Ana Atti, Russell Hope, Julie Robotham, Susan Hopkins, Colin S Brown, Victoria J Hall, SIREN Study Group, Omoyeni Adebiyi, David Adeboyeku, Jonnie Aeron-Thomas, Masood Aga, K Agwuh, S Ahmad, Talat Akhtar, S Akhtar, N Aldridge, Zehra’a Al-Khafaji, Lynne Allsop, Shrikant Ambalkar, Nick Andrews, Alejandro Arenas-Pinto, Helen Ashby, John Ashcroft, Maxine Ashton, Cressida Auckland, Manny Bagary, Sarah Baillon, Steve Bain, Philippa Bakker, Lisa Barbour, T Barnes, Vhairi Bateman, Mandy Beekes, L Berry, Janki Bhayani, Jennifer Bishop, Lisa Bishop, Karen Black, Sarah Board, David Boss, Angel Boulos, G Boyd, Simon Brake, Sarah Brand, Andrew Broadley, Claire Brookes, Tim Brooks, Alison Brown, Kerryanne Brown, D Browne, Philippa Burns, Ben Burton, Georgina Butt, Davina Calbraith, Euan Cameron, Meera Chand, Badrinathan Chandrasekaran, Andre Charlett, Ray Chaudhuri, Anu Chawla, H Chenoweth, Nihil Chitalia, Alexandra Cochrane, Louise Coke, Michelle Cole, Holly Coles, James Colton, Joanna Conneely, Paul Conneely, Claire Corless, Dianne Corrigan, Kathryn Court, Anna Cowley, Peter Cowling, Lisa Cromey, Roxanne Crosby-Nwaobi, Jane Crowe, Silvia D’Arcangelo, Ananta Dave, Joy Dawson, Ellen de Lacy, Thushan de Silva, Ismaelette Del Rosario, Jane Democratis, Devesh Dhasmana, Stephanie Diaz, Allison Dimmer, Lisa Ditchfield, Laura Dobbie, Allison Doel, Tracy Donaghy, Christopher Duff, David Dytmer, Nicholas Easom, Joanne Edgar, Tracy Edmunds, John Elliott, Yvette Ellis, Ngozi Elumogo, Eve Etell Kirby, Barzo Faris, Clair Favager, Lauren Finlayson, Christine Ford, Susan Fowler, Nabila Fowles-Gutierrez, Eva Fraile, Joan Frieslick, Susannah Froude, Eileen Gallagher, Joanne Galliford, F Game, John Geen, Hannah Gibson, J Giles, David Goldberg, Jayne Goodwin, S Gormley, Imogen Gould, Alison Grant, Jennifer Graves, Kim Gray, Joanne Gray, Katherine Gray, Marie Green, Susan Greenwood, Simantee Guha, Christian Hacon, Mathew Halkes, Steve Hams, Esther Hanison, Elinor Hanna, Pratap Harbham, Paula Harman, Edward Harris, Penny Harris, G Harrison, D Harvey, J Harwood, Jonathan Hatton, April Hawkins, Nipunadi Hettiarachchi, Jacqueline Hewson, Andrew Higham, Sarah Hinch, Antonia Ho, Ina Hoad, Kyra Holliday, Kathryn Hollinshead, Christopher Holmes, Stacey Horne, A Horsley, Angela Houston, M Howard, Joanne Howard, Deborah Howcroft, Kate Howell, Y Huang, Karen Hudson, Lauren Hughes, Ferdinando Insalata, Val Irvine, Kate James, Frances Johnston, Helen Johnstone, C Jones, Annelysse Jorgenson, Hannah Jory, Maya Joseph, Nagesh Kalakonda, Philippa Kemsley, Siobhan Keogh, C Kerrison, Shivani Khan, Sheena Khanduri, Jameel Khawam, Robert Kyffin, A Larru, Scott Latham, Richard Laugharne, Rajeka Lazarus, Mihye Lee, Yvonne Lester, Kenisha Lewis, T Lewis, Tracy Lewis, Ezra Linley, Josie Long, Ruth Longfellow, Clodagh Loughrey, Murray Luckas, Shekoo Mackay, Natasha Mahabir, Tabitha Mahungu, Martin Malcolm, Diego Maseda, Ayo Matuluko, V Maxwell, Clare McAdam, Danielle McCracken, S Mcwilliam, Manjula Meda, Pauline Mercer, Iain Milligan, Mariyam Mirfenderesky, Raksha Mistry, A Moody, Kelly Moran, Caroline Mulvaney Jones, Katie Munro, Michael Murphy, Maxine Nash, Claire Neill, K Nimako, Chris Norman, Chris Norman, Anne-Marie O’Connell, Maurice O'Kane, A O'Kelly, Zohra Omar, Ashley Otter, Kitty Paques, Manish Patel, B Payne, C Pegg, R Penn, Stacey Pepper, Justin Pepperell, James Pethick, Graham Pickard, Charlie Piercy, Tim Planche, Aiden Plant, Carla Pothecary, G Pottinger, James Powell, Lesley Price, Cathy Price, Stephanie Prince, Rowan Pritchard Jones, Yuri Protaschik, Zaman Qazzafi, A Rajgopal, Mary Ramsay, Chloe Reeks, T Reynolds, Lisa Richardson, P Ridley, Julia Roberts, L Robinson, Alison Rodger, Alun Roebuck, Anna Rokakis, Cathy Rowe, Anna Roynon, J Russell, Lauren Sach, Ayoub Saei, Noshin Sajedi, Amanda Semper, Nicola Sergenson, Abigail Severn, A Shah, K Shipman, Robert Shorten, R Sierra, Janet Sinclair, Catherine Sinclair, Aaran Sinclair, Helena Sovriarova, Claire Stafford, Guy Stevens, B Stewart, Sophia Strong-Sheldrake, Banerjee SubhroOsuji, Mags Szewczyk, Esther Tarr, Andrew Taylor-Kerr, Simon Tazzyman, Andrew Telfer, Rebecca Temple-Purcell, Kate Templeton, Claire Thomas, Catherine Thompson, Fiona Thompson, R Tilley, Jean Timeyin, Anne Todd, Johanne Tomlinson, Simon Tonge, Caio Tranquillini, Tom Trinick, Linda Tyson, Julia Vasant, Neringa Vilimiene, Lynne Walker, Emma Ward, Simon Warren, Ekaterina Watson, A Watt, Jennifer Weir, Fran Westwell, Amanda Whileman, Nikki White, Beverly Wilkinson, Claire Williams, M Williams, Stephanie Willshaw, E Wilson-Davies, Stephen Winchester, Martin Wiselka, N Wong, Megan Woolcook, Diane Wycherley, Charlotte Young, Maria Zambon, Qi Zheng

**Affiliations:** 1 UK Health Security Agency, London, UK; 2 The National Institute for Health Research Health (NIHR) Protection Research Unit in Healthcare Associated Infections and Antimicrobial Resistance at the University of Oxford, Oxford, UK; 3 The National Institute for Health Research (NIHR) Health Protection Research Unit in Healthcare Associated Infections and Antimicrobial Resistance at Imperial College London, London, UK; * Joint first authors: contributed equally

## Abstract

**Objective:**

To describe the incidence of, risk factors for, and impact of vaccines on primary SARS-CoV-2 infection during the second wave of the covid-19 pandemic in susceptible hospital healthcare workers in England.

**Design:**

Multicentre prospective cohort study.

**Setting:**

National Health Service secondary care health organisations (trusts) in England between 1 September 2020 and 30 April 2021.

**Participants:**

Clinical, support, and administrative staff enrolled in the SARS-CoV-2 Immunity and Reinfection Evaluation (SIREN) study with no evidence of previous infection. Vaccination status was obtained from national covid-19 vaccination registries and self-reported.

**Main outcome measure:**

SARS-CoV-2 infection confirmed by polymerase chain reaction. Mixed effects logistic regression was conducted to determine demographic and occupational risk factors for infection, and an individual based mathematical model was used to predict how large the burden could have been if vaccines had not been available from 8 December 2020 .

**Results:**

During England’s second wave, 12.9% (2353/18 284) of susceptible SIREN participants became infected with SARS-CoV-2. Infections peaked in late December 2020 and decreased from January 2021, concurrent with the cohort’s rapid vaccination coverage and a national lockdown. In multivariable analysis, factors increasing the likelihood of infection in the second wave were being under 25 years old (20.3% (132/651); adjusted odds ratio 1.35, 95% confidence interval 1.07 to 1.69), living in a large household (15.8% (282/1781); 1.54, 1.23 to 1.94, for participants from households of five or more people), having frequent exposure to patients with covid-19 (19.2% (723/3762); 1.79, 1.56 to 2.06, for participants with exposure every shift), working in an emergency department or inpatient ward setting (20.8% (386/1855); 1.76, 1.45 to 2.14), and being a healthcare assistant (18.1% (267/1479); 1.43, 1.16 to 1.77). Time to first vaccination emerged as being strongly associated with infection (P<0.001), with each additional day multiplying a participant’s adjusted odds ratio by 1.02. Mathematical model simulations indicated that an additional 9.9% of all patient facing hospital healthcare workers would have been infected were it not for the rapid vaccination coverage.

**Conclusions:**

The rapid covid-19 vaccine rollout from December 2020 averted infection in a large proportion of hospital healthcare workers in England: without vaccines, second wave infections could have been 69% higher. With booster vaccinations being needed for adequate protection from the omicron variant, and perhaps the need for further boosters for future variants, ensuring equitable delivery to healthcare workers is essential. The findings also highlight occupational risk factors that persisted in healthcare workers despite vaccine rollout; a greater understanding of the transmission dynamics responsible for these is needed to help to optimise the infection prevention and control policies that protect healthcare workers from infection and therefore to support staffing levels and maintain healthcare provision.

**Trial registration:**

ISRCTN registry ISRCTN11041050.

## Introduction

In autumn 2020 England entered its second wave of the SARS-CoV-2 pandemic. During this eight month period (1 September 2020 to 30 April 2021), the National Health Service (NHS) was under considerable strain, with covid-19 related admissions peaking at 25 938 during the week of 4 January 2021.^1^ The most intense period of the second wave followed the lifting of England’s second national lockdown on 2 December 2020,^1^ ^2^ and it was likely amplified by the emergence and spread of the more transmissible alpha variant (B.1.1.7).^3^ ^4^ Fortunately, this period also saw the delivery of the UK’s first licensed covid-19 vaccine (Pfizer-BioNTech: 8 December 2020), with further vaccines introduced shortly after (AstraZeneca: 4 January 2021; Moderna: 7 April 2021).^5^ ^6^ A rapid vaccine rollout across England followed, prioritising frontline healthcare workers and achieving high population coverage.^7^ ^8^

During the first wave, hospital healthcare workers were observed to be at higher risk of exposure to and infection with SARS-CoV-2 than the general population.^9^ ^10^ ^11^ ^12^ Demographic characteristics, such as ethnicity, and occupational factors, such as occupational setting, shift work, and frequency of exposure to patients with covid-19, were associated with higher risk.^13^ ^14^ ^15^ ^16^ ^17^ ^18^ ^19^ Understanding how these factors contribute to infection risk within the healthcare workforce is essential for policy planning, especially in the context of emerging variants of concern, such as the omicron variant (B.1.1.529), which may require vaccine boosters for adequate protection against primary infection and reinfection.^20^ ^21^ ^22^ ^23^ ^24^ ^25^ ^26^ ^27^ Staff shortages due to illness and isolation, particularly during the peak of the second wave before the vaccine rollout, compounded the already high clinical burden faced by the NHS in winter 2020-21.^28^ ^29^ Protecting healthcare workers from infection is crucial to not only their health but also healthcare provision and the safety of patients.

In this study, we aimed to describe the incidence of, risk factors for, and impact of vaccines on primary SARS-CoV-2 infection during the second wave of the pandemic in a large cohort of susceptible healthcare workers in England, enrolled into the SARS-CoV-2 Immunity and Reinfection Evaluation (SIREN) study. Our findings are relevant to inform hospital infection prevention and control policy for healthcare workers if further waves of the SARS-CoV-2 pandemic occur and to guide future winter pressure preparedness in the NHS, particularly if SARS-CoV-2 begins to contribute annually.

## Methods

### Study design and participants

SIREN is a multicentre prospective cohort study among NHS staff in the UK across 135 healthcare organisations, investigating immunity to SARS-CoV-2 following infection and vaccination. In England, SIREN centres represent secondary care health organisations (NHS trusts) that can operate over several hospital sites. The full study design and methods have been described previously.^30^ ^31^

Our study population was SIREN participants who entered the second wave susceptible to a primary SARS-CoV-2 infection, defined as having no record of SARS-CoV-2 polymerase chain reaction (PCR) or serological positivity. We included participants in our statistical analysis if they were susceptible to primary infection on 1 September 2020 or on enrolment date, if enrolled between 1 September 2020 and 7 December 2020 (delayed cohort entry). We excluded participants from this analysis if they enrolled after the start of the vaccine rollout (8 December 2020) or did not have documented and linked PCR tests during the second wave, defined here as 1 September 2020 to 30 April 2021.

We restricted our analysis to participants recruited from English sites only to match source datasets for the modelling component of this study (appendix 1: mathematical modelling methods). For our risk factor (regression) analysis, we removed participants who could not contribute to all demographic, household, and occupational variables (missing gender, ethnicity, household, or postcode details).

### Data collection and sources

Participants underwent fortnightly asymptomatic PCR testing and monthly antibody testing at their site of enrolment, as per protocol.^31^ PCR and serology assay type and threshold for positivity varied according to local laboratory protocols. In addition, frontline healthcare workers were able to participate in twice weekly lateral flow device testing (with PCR confirmation of positive lateral flow device results), as per government guidelines and hospital policy.^32^ Data on demographics and exposures (workplace, community, and household) were collected in the enrolment questionnaire.

The SIREN database comprises all SARS-CoV-2 PCR and serology results captured by the UK Health Security Agency (UKHSA) Second Generation Surveillance System (SGSS) since the beginning of the pandemic, whether taken clinically or as part of SIREN, and questionnaire results. We obtained participants’ vaccination data by questionnaire and linkage on personal identifiable information to the National Immunisation Management System (NIMS).

### Outcome

We used primary infection, defined as the first PCR positive result of a susceptible participant, as the primary outcome of our statistical analyses. We defined the date of infection as the specimen date of the sample.

### Statistical analysis

We calculated the weekly incidence of primary SARS-CoV-2 infection and the weekly cumulative vaccination coverage (one or more doses) in SIREN participants susceptible to primary infection both nationally and regionally. We also estimated the weekly vaccination coverage for demographic and occupational subgroups. We used R version 4.1.1 for these analyses.

We stratified primary infection attack rates during the second wave by demographic, household, and occupational characteristics (gender, age group, ethnicity, medical conditions, Index of Multiple Deprivation fifth, household size, children in household, region, frequency of exposure to patients with covid-19, occupational setting, and occupation). We calculated odds ratios and adjusted odds ratios for primary infection: adjustment used a mixed effects logistic regression model, reported with 95% confidence intervals and Wald test results. In this regression, all stratification characteristics were included as categorical variables (fixed effects) within organisation level clusters (random effects). We included continuous fixed effect variables to adjust for time contributing to the analysis (owing to the rolling recruitment to the cohort) and for time to first vaccination. We used Stata Statistical Software (release 15.1) for these analyses.

### Mathematical model

We assessed the impact of the vaccination programme on infection rates by using a mathematical model, with which we simulated a counterfactual scenario in which nobody was vaccinated. We compared this with modelled output for the scenario representing the vaccine rollout in England. This individual based model simulated transmission between and within patient facing hospital healthcare workers and patients. Full details and parameterisation of the modelling methods are given in appendix 1: mathematical modelling methods.

In summary, the model had a susceptible-exposed-infected-recovered (SEIR) structure: simulated individuals represented patient facing hospital healthcare workers and patients, all of whom were all fully susceptible at the start of pandemic, and who progressed on transmission through an incubation period before an infectious stage (including asymptomatic cases) and recovery. A schematic of the model is presented in appendix 2: supplementary figure C. The individual based model simulated transmission to patient facing hospital healthcare workers via three routes: from the public (community), from patients in hospital, and from other patient facing hospital healthcare workers. Nosocomial transmission to patients could occur via three routes: direct transmission to susceptible patients from infected patients, direct transmission from infected patient facing hospital healthcare workers (for example, by droplet or aerosol), and indirect transmission from infected patients (for example, via fomites). We included change in vaccination status by using a logistic growth curve fitted to data from SIREN-NIMS linked data (for patient facing hospital healthcare workers) and NIMS data (for the public). Vaccine efficacy was included from 21 days after the first dose, reducing transmission (by 50%) and preventing infection (at 70% efficacy, rising to 80% after two doses).^33^ ^34^ ^35^ The virulence of each variant circulating throughout the second wave and the probability of an individual having each variant were included by using previously published NHS regional data.^36^

The individual based model was calibrated using cumulative infection data from the SIREN study between 1 March 2020 and 8 December 2020, and from NHS Situational Reports (appendix 2: supplementary figure D).^37^

### Participant and public involvement

As part of the SIREN study, we have engaged with our participants throughout via regular newsletters and webinars. More recently, we have established a participant and public involvement panel that will meet every six weeks to ensure that the research we generate remains relevant. The findings of this work will be discussed at the next webinar and participant and public involvement working groups.

## Results

**Fig 1:**
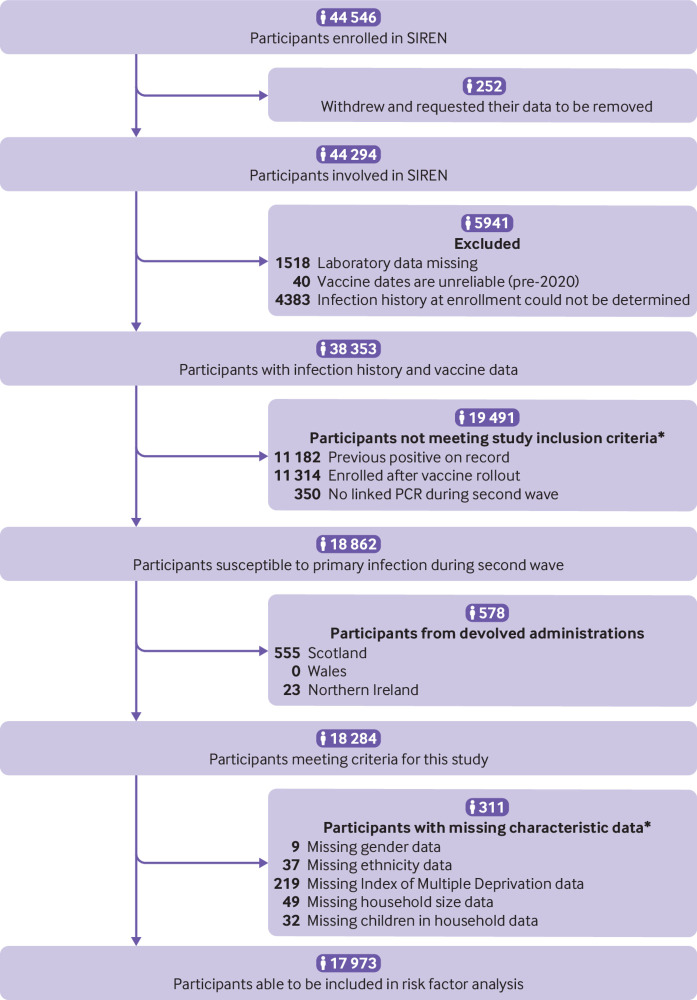
Flow diagram of participant inclusion and exclusion criteria used to define study cohorts for analysis. Crude attack rates by groups of susceptible participants excluded from risk factor analysis (devolved administrations (n=578) and missing data (n=311)) are available in supplementary tables A and B. PCR=polymerase chain reaction. *Participants may meet more than one exclusion criterion

**Fig 2:**
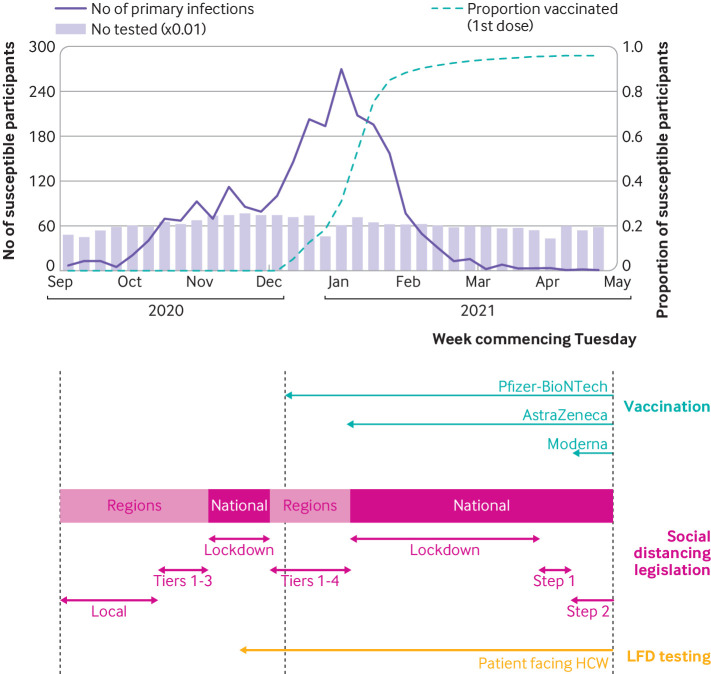
Weekly incidence of SARS-CoV-2 primary infections, and weekly cumulative vaccination coverage, in SIREN participants susceptible to primary infection in England, with calendar of England-wide covid-19 interventions during second wave (1 September 2020 to 30 April 2021). HCW=healthcare workers; LFD=lateral flow device

**Fig 3:**
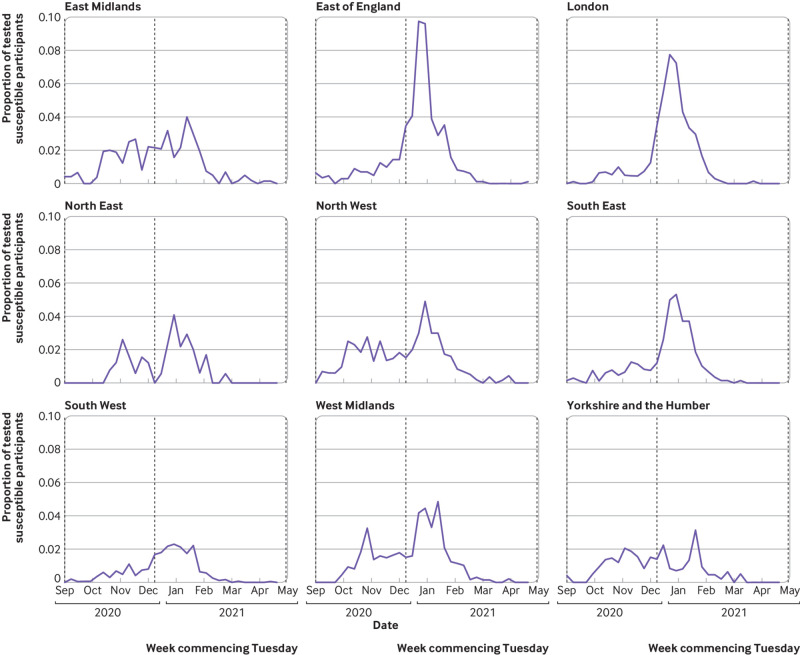
Positive polymerase chain reaction results as proportion of SIREN participants susceptible to primary infection in England tested by week, stratified by region (1 September 2020 to 30 April 2021)

**Fig 4:**
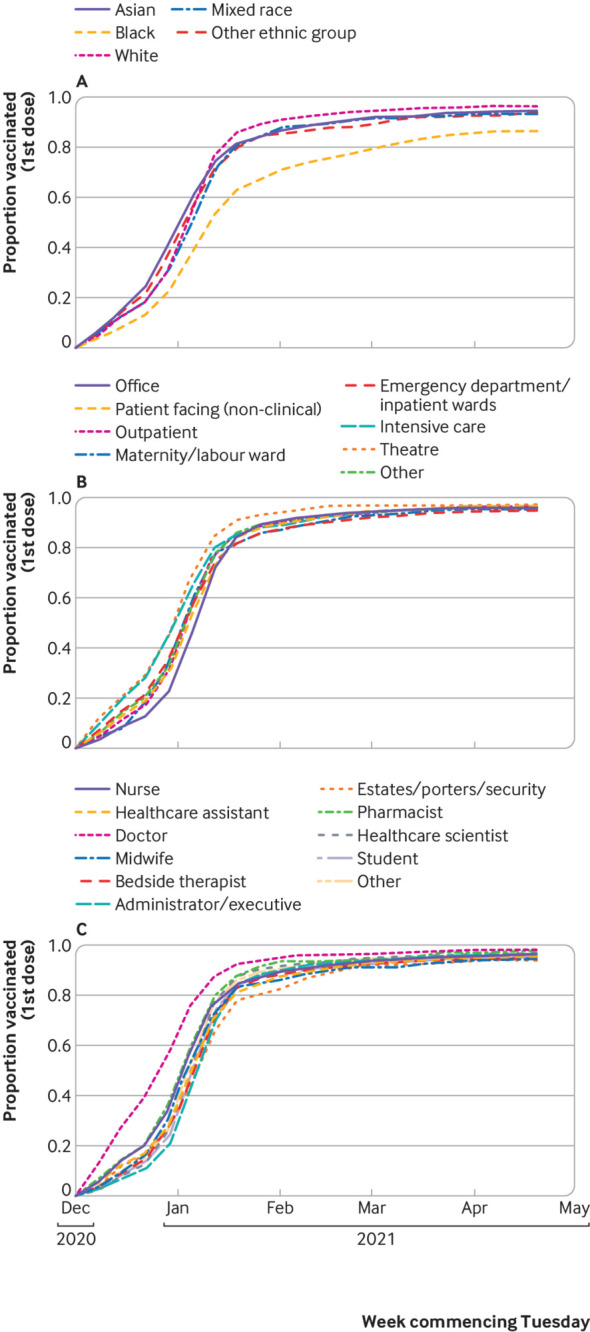
Weekly cumulative vaccination coverage of SIREN participants susceptible to primary infection in England, stratified by demographic and occupational characteristics. Proportion of susceptible SIREN participants with complete characteristic data (n=17 973) and one or more dose of covid-19 vaccine, by date of first dose, stratified by ethnicity (A), occupational setting (B), and occupation (C)

We included 18 284 susceptible participants from England, recruited from 105 secondary care health organisations (fig 1). Table 1 shows their demographic, household, and occupational characteristics. Between the beginning of September 2020 and the end of April 2021, 2353 new primary infections occurred: a crude attack rate of 12.9%. Figure 2 shows the weekly incidence of primary infection in this cohort. Incidence peaked during the week of 29 December 2020 and then rapidly decreased, coinciding with vaccine rollout and England’s third national lockdown beginning on 6 January 2021 (fig 2). Vaccination coverage (first dose) was 26.9% (4914/18 284) on 31 December 2021, 88.3% (16 143/18 284) on 31 January 2021, and 96.1% (17 576/18 284) on 30 April 2021. The peak incidence was highest in the East of England, London, and South East regions (fig 3), which all had the earliest rise in circulation of the alpha variant in the community (appendix 2: supplementary figure A).^38^ Rate of initial growth in vaccination coverage (26.8% (4825/17 973) by 31 December 2020) varied by occupational factors and ethnicity (fig 4). It was fastest among doctors (50.4% (923/1832); P<0.001 for proportion comparison) and staff working in intensive care settings (41.2% (417/1012); P<0.001) or in theatres (38.9% (124/319); P<0.001). Growth in vaccination coverage was slowest in administrators (15.9% (464/2917); P<0.001), office based staff (18.6% (736/3953); P<0.001), and participants of black ethnicity (19.4% (55/284); P=0.006).

**Table 1:** Demographic, household, and occupational characteristics of SIREN participants susceptible to primary infection in second wave of SARS-CoV-2 in England. Values are numbers (percentages)

Characteristic	Susceptible to primary infection during second wave (n=18 284)
Gender:	
Female	15 528 (84.9)
Male	2730 (14.9)
Non-binary	17 (0.1)
Prefer not to say	9 (0.0)
Age group:	
<25	675 (3.7)
25-34	3566 (19.5)
35-44	4544 (24.9)
45-54	5555 (30.4)
55-64	3627 (19.8)
≥65	317 (1.7)
Ethnicity:	
Asian	1207 (6.6)
Black	297 (1.6)
White	16 238 (88.8)
Mixed race	296 (1.6)
Other ethnic group	209 (1.1)
Prefer not to say	37 (0.2)
Medical conditions:	
No medical conditions	13 595 (74.4)
Immunosuppression	402 (2.2)
Chronic respiratory disease	2365 (12.9)
Chronic non-respiratory disease	1922 (10.5)
Index of Multiple Deprivation fifth*:	
1 (most deprived)	1936 (10.6)
2	3243 (17.7)
3	4216 (23.1)
4	4341 (23.7)
5 (least deprived)	4329 (23.7)
Unknown	219 (1.2)
Household size:	
1	1828 (10.0)
2	6068 (33.2)
3 or 4	8535 (46.7)
≥5	1804 (9.9)
Prefer not to say	49 (0.3)
Children in household:	
No children	10 780 (59.0)
Children	7472 (40.9)
Prefer not to say	32 (0.2)
Region:	
East Midlands	1803 (9.9)
East of England	2263 (12.4)
London	2065 (11.3)
North East	373 (2.0)
North West	1976 (10.8)
South East	2398 (13.1)
South West	4182 (22.9)
West Midlands	1791 (9.8)
Yorkshire and the Humber	1433 (7.8)
Frequency of close proximity to patients with covid-19:	
Every shift	3833 (21.0)
Once a week	3050 (16.7)
Once a month	1651 (9.0)
Less than once a month	2646 (14.5)
Never	7104 (38.9)
Occupational setting:	
Office	4002 (21.9)
Patient facing (non-clinical)	687 (3.8)
Outpatient	3273 (17.9)
Maternity/labour ward	193 (1.1)
Emergency department†/inpatient ward	1906 (10.4)
Intensive care	1029 (5.6)
Theatres	324 (1.8)
Other	6870 (37.6)
Occupation:	
Nurse	6011 (32.9)
Healthcare assistant	1497 (8.2)
Doctor	1890 (10.3)
Midwife	465 (2.5)
Bedside therapist‡	572 (3.1)
Administrator/executive (office based)	2953 (16.2)
Estates/porters/security	170 (0.9)
Pharmacist	280 (1.5)
Healthcare scientist	702 (3.8)
Student§	909 (5.0)
Other	2835 (15.5)

* Residential relative deprivation score according to postcode and English Indices of Deprivation 2019.

† Including ambulance setting

‡ Physiotherapist, occupational therapist, speech and language therapist.

§ Medical student, nursing student, midwifery student, student: other.

### Risk factors for infection in SIREN participants

Our regression analyses included 17 973 participants who met our criteria (fig 1). Table 2 shows analyses of potential demographic and household risk factors for primary infection, and table 3 shows occupational risk factors. Table 3 also shows the temporal variables included in the regression model.

**Table 2:** Association of demographic and household characteristics of susceptible SIREN participants with primary infection during second wave of SARS-CoV-2 pandemic in England (1 September 2020 to 30 April 2021)

Characteristic	Infected (n=2318)	Total* (n=17 973)	Infected (%)	Odds ratio (95% CI)	P value	Adjusted odds ratio† (95% CI)	P value
Gender†:							
Female	1950	15 290	12.8	Reference	-	Reference	-
Male	365	2665	13.7	1.08 (0.96 to 1.22)	0.21	1.16 (1.01 to 1.33)	0.03
Non-binary‡	-	≤17	-	1.05 (0.24 to 4.66)	0.95	1.00 (0.20 to 4.90)	01.00
Age group:							
<25	132	651	20.3	1.86 (1.51 to 2.29)	<0.001	1.35 (1.07 to 1.69)	0.01
25-34	540	3455	15.6	1.35 (1.20 to 1.53)	<0.001	0.94 (0.82 to 1.08)	0.42
35-44	555	4486	12.4	1.03 (0.92 to 1.16)	0.60	0.90 (0.79 to 1.02)	0.10
45-54	659	5479	12.0	Reference	-	Reference	-
55-64	406	3591	11.3	0.93 (0.82 to 1.06)	0.30	1.00 (0.87 to 1.15)	0.98
≥65	26	311	8.4	0.67 (0.44 to 1.01)	0.05	0.73 (0.48 to 1.12)	0.15
Ethnicity:							
Asian	211	1163	18.1	1.57 (1.34 to 1.84)	<0.001	1.23 (1.03 to 1.47)	0.02
Black	65	284	22.9	2.10 (1.59 to 2.79)	<0.001	1.18 (0.86 to 1.62)	0.30
White	1983	16 034	12.4	Reference	-	Reference	-
Mixed race	33	288	11.5	0.92 (0.64 to 1.32)	0.64	0.72 (0.49 to 1.06)	0.10
Other ethnic group	26	204	12.7	1.03 (0.68 to 1.57)	0.87	0.73 (0.47 to 1.13)	0.16
Medical conditions:							
No medical conditions	1736	13 355	13.0	Reference	-	Reference	-
Immunosuppression	38	396	9.6	0.71 (0.51 to 1.00)	0.05	0.75 (0.52 to 1.06)	0.10
Chronic respiratory disease	290	2332	12.4	0.95 (0.83 to 1.09)	0.45	1.01 (0.88 to 1.16)	0.90
Chronic non-respiratory disease	254	1890	13.4	1.04 (0.90 to 1.20)	0.60	1.11 (0.96 to 1.29)	0.16
Index of Multiple Deprivation fifth:							
1 (most deprived)	302	1922	15.7	1.36 (1.17 to 1.59)	<0.001	1.05 (0.88 to 1.25)	0.58
2	430	3220	13.4	1.13 (0.98 to 1.29)	0.09	1.04 (0.89 to 1.20)	0.64
3	510	4200	12.1	1.01 (0.89 to 1.15)	0.89	0.99 (0.86 to 1.14)	0.90
4	557	4323	12.9	1.08 (0.95 to 1.23)	0.24	1.05 (0.92 to 1.20)	0.48
5 (least deprived)	519	4308	12.0	Reference	-	Reference	-
Household size:							
1	189	1801	10.5	Reference	-	Reference	-
2	755	5971	12.6	1.23 (1.04 to 1.46)	0.01	1.21 (1.01 to 1.44)	0.04
3 or 4	1092	8420	13.0	1.27 (1.08 to 1.50)	0.004	1.32 (1.09 to 1.59)	0.004
≥5	282	1781	15.8	1.60 (1.32 to 1.96)	<0.001	1.54 (1.23 to 1.94)	<0.001
Children in household:							
Children	1357	10 592	12.8	Reference	-	Reference	-
No children	961	7381	13.0	1.02 (0.93 to 1.11)	0.68	0.92 (0.81 to 1.05)	0.23
Region:							
East Midlands	265	1775	14.9	1.87 (1.58 to 2.22)	<0.001	1.66 (1.18 to 2.32)	0.003
East of England	375	2233	16.8	2.15 (1.84 to 2.52)	<0.001	1.92 (1.41 to 2.60)	<0.001
London	316	1990	15.9	2.01 (1.71 to 2.37)	<0.001	1.71 (1.27 to 2.31)	<0.001
North East	41	366	11.2	1.34 (0.95 to 1.89)	0.090	1.48 (0.81 to 2.69)	0.20
North West	309	1956	15.8	2.00 (1.70 to 2.36)	<0.001	1.91 (1.42 to 2.57)	<0.001
South East	267	2364	11.3	1.36 (1.15 to 1.60)	<0.001	1.49 (1.12 to 1.98)	0.006
South West	352	4102	8.6	Reference	-	Reference	-
West Midlands	256	1776	14.4	1.79 (1.51 to 2.13)	<0.001	1.68 (1.23 to 2.29)	0.001
Yorkshire and the Humber	137	1411	9.7	1.15 (0.93 to 1.41)	0.20	1.15 (0.81 to 1.62)	0.43

* 311 participants excluded from risk factor analyses owing to missing characteristics data.

† Adjusted for above characteristics and those in table 3, with secondary care health organisation as random effect.

‡ Owing to low absolute number of infections in participants identifying as non-binary, counts within gender categories have either been suppressed or rounded to nearest 5 to mitigate disclosure risk.

**Table 3:** Association of occupational and temporal characteristics of susceptible SIREN participants with primary infection during second wave of SARS-CoV-2 pandemic in England (1 September 2020 to 30 April 2021)

Characteristic	Infected (n=2318)	Total* (n=17 973)	Infected (%)	Odds ratio (95% CI)	P value	Adjusted odds ratio† (95% CI)	P value
Frequency of close proximity to patients with covid-19:							
Every shift	723	3762	19.2	2.36 (2.10 to 2.65)	<0.001	1.79 (1.56 to 2.06)	<0.001
Once a week	436	2981	14.6	1.70 (1.49 to 1.93)	<0.001	1.45 (1.25 to 1.68)	<0.001
Once a month	219	1620	13.5	1.55 (1.31 to 1.82)	<0.001	1.39 (1.16 to 1.66)	<0.001
Less than once a month	298	2609	11.4	1.28 (1.10 to 1.48)	0.001	1.19 (1.02 to 1.40)	0.03
Never	642	7001	9.2	Reference	-	Reference	-
Occupational setting:							
Office	393	3953	9.9	Reference	-	Reference	-
Patient facing (non-clinical)	93	676	13.8	1.45 (1.13 to 1.84)	0.003	1.24 (0.94 to 1.63)	0.12
Outpatient	406	3223	12.6	1.31 (1.13 to 1.51)	<0.001	1.02 (0.86 to 1.22)	0.80
Maternity/labour ward	18	189	9.5	0.95 (0.58 to 1.57)	0.85	0.87 (0.51 to 1.49)	0.61
Emergency department‡/inpatient wards	386	1855	20.8	2.38 (2.04 to 2.78)	<0.001	1.76 (1.45 to 2.14)	<0.001
Intensive care	135	1012	13.3	1.39 (1.13 to 1.72)	0.002	0.90 (0.71 to 1.14)	0.39
Theatres	31	319	9.7	0.98 (0.66 to 1.43)	0.90	0.80 (0.52 to 1.22)	0.30
Other	856	6746	12.7	1.32 (1.16 to 1.49)	<0.001	1.06 (0.91 to 1.23)	0.47
Occupation:							
Nurse	821	5918	13.9	1.43 (1.24 to 1.64)	<0.001	1.12 (0.94 to 1.33)	0.20
Healthcare assistant	267	1479	18.1	1.95 (1.63 to 2.34)	<0.001	1.43 (1.16 to 1.77)	0.001
Doctor	222	1832	12.1	1.22 (1.01 to 1.47)	0.03	0.99 (0.78 to 1.24)	0.91
Midwife	46	458	10.0	0.99 (0.71 to 1.37)	0.95	0.76 (0.53 to 1.10)	0.15
Bedside therapist§	96	556	17.3	1.85 (1.44 to 2.38)	<0.001	1.32 (0.99 to 1.76)	0.06
Administrator/executive (office based)	296	2917	10.1	Reference	-	Reference	-
Estates/porters/security	31	170	18.2	1.97 (1.31 to 2.97)	<0.001	1.34 (0.86 to 2.10)	0.20
Pharmacist	34	270	12.6	1.28 (0.87 to 1.86)	0.21	0.89 (0.58 to 1.37)	0.61
Healthcare scientist	55	689	8.0	0.77 (0.57 to 1.04)	0.09	0.73 (0.53 to 1.01)	0.06
Student¶	110	894	12.3	1.24 (0.98 to 1.57)	0.07	1.00 (0.78 to 1.29)	0.10
Other	340	2790	12.2	1.23 (1.04 to 1.45)	0.01	1.02 (0.84 to 1.23)	0.87
Temporal characteristics:							
Time to vaccination (days after 8 December)	-	-	-	-	-	1.02 (1.01 to 1.02)	<0.001
Time in cohort (days)	-	-	-	-	-	1.00 (0.99 to 1.00)	<0.001

* 311 participants excluded from risk factor analyses owing to missing characteristics data.

† Adjusted for above characteristics and those in table 2, with secondary care health organisation as random effect.

‡ Including ambulance setting.

§ Physiotherapist, occupational therapist, speech and language therapist.

¶ Medical student, nursing student, midwifery student, student: other.

Within demographic and household characteristics, we observed the highest infection rates in younger participants, participants of Asian or black ethnicity, and participants living in larger households (table 2). Within occupational characteristics, the highest infection rates were in participants working in settings where they were often exposed to patients with covid-19, participants who worked in emergency department or inpatient ward settings, and participants working as healthcare assistants, bedside therapists, porters, and security and estates staff (table 3).

Before adjustment, univariate analyses suggested that many demographic, household, and occupational characteristics had a significant association with infection, in addition to those with the highest crude infection rates (table 2; table 3). However, after adjustment, which included time to first vaccination as a strongly associated predictor (P<0.001, with each additional day multiplying a participant’s adjusted odds ratio by 1.02), many of these associations were weakened. Characteristics that remained a strong risk (at P≤0.01) after adjustment were being under 25 years old, living in a household of three or more people, having exposure to patients with covid-19 at least once monthly, working in an emergency department or inpatient ward setting, and being a healthcare assistant.

We observed regional differences, which remained after adjustment, with the greatest adjusted odds ratios being for participants working either in the highest peak regions or in the Midlands and North West (where incidence in the autumn was above average). The factors included in the regression model accounted for 54% of the variation seen between organisation level clusters: the median second wave attack rate by organisation was 12.5% (interquartile range 8.9-15.9%; n=92 organisations with study sample >50).

### Estimated impact of vaccines on patient facing hospital healthcare workers

**Fig 5:**
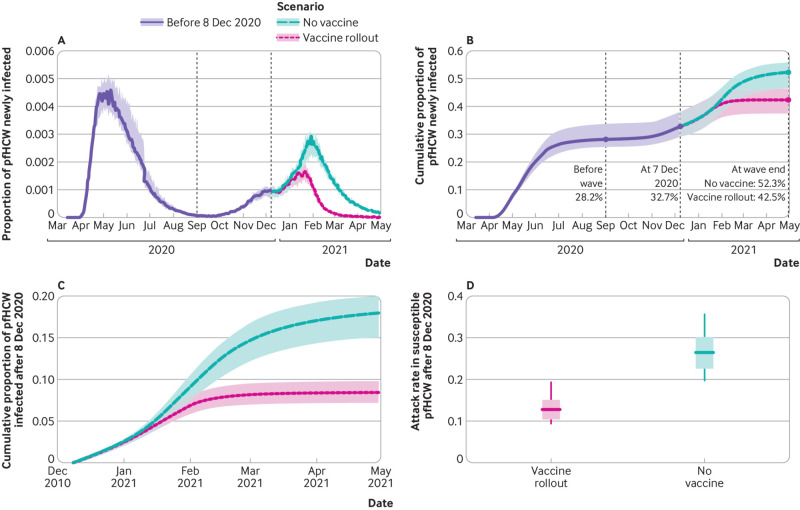
Effect of vaccines on rates of SARS-CoV-2 infection in patient facing hospital healthcare workers (pfHCW) from simulation output. Individual based model simulation output for “vaccine rollout” and “no vaccines” scenarios. A) Proportion of pfHCW infected per day until 30 April 2021. B) Daily cumulative proportion of pfHCW infected until 30 April 2021. C) Daily cumulative proportion of pfHCW infected after 8 December 2020 (start of vaccine rollout). D) Proportion of susceptible pfHCW at 8 December 2020 who were subsequently infected by end of April 2021

Simulated infection rates in patient facing hospital healthcare workers under a “vaccine rollout” scenario, in which vaccination proceeded as observed via NIMS, began to diverge from a counterfactual “no vaccines available” scenario after 15 January 2021 (fig 5). Allowing for the latent period of SARS-CoV-2 infection, these cases would not be detectable by PCR until two to 14 days later. In the model, 52.3% (interquartile range 46.6-55.9%) of patient facing hospital healthcare workers were infected by the end of April 2021 in the counterfactual no vaccines available scenario simulations compared with 42.5% (37.5-46.4%) in the vaccine rollout scenario; this equates to a 19% cut in cumulative infections from the start of the pandemic due to vaccine rollout. As 28.2% (25.4-33.7%) of simulated patient facing hospital healthcare workers had been infected before the start of the second wave, the model results indicate that infections in patient facing hospital healthcare workers during the second wave would have been 69% (24.1/14.3) higher had vaccines not been available.

After 8 December 2020, the total attack rate in susceptible patient facing hospital healthcare workers in the counterfactual scenario simulations was twice as high as in the vaccine rollout scenario (fig 5). In these simulations, the greatest absolute effect of vaccines was seen in East of England, where 16.7% fewer patient facing hospital healthcare workers were infected between 8 December 2020 and 30 April 2021 (33.4% (interquartile range 29.9-38.9%) versus 17.1% (14.2-20.4%)), and the smallest absolute effect was seen in South West (9.5% difference: 17.8% (16.3-19.8%) versus 8.3% (7.0-9.1%)) (appendix 2: supplementary figures E and F). This reflects the timing of the alpha variant’s circulation in those regions (appendix 2: supplementary figure C).

## Discussion

During the eight months of England’s second wave of SARS-CoV-2, 12.9% of SIREN participants susceptible to primary infection became infected. From our mathematical model, we estimate that an additional 9.9% of all patient facing hospital healthcare workers would have been infected during this period were it not for the rapid vaccination coverage, prioritising this group.

After peaking in late December 2020, new primary infections decreased sharply, concurrent with the rapid vaccination coverage in the cohort and a national lockdown. We found the strongest risk factor for infection in the second wave to be time to first vaccination: disparities in vaccination coverage within our cohort are likely to account for the strong univariate association of infection risk with black ethnicity disappearing after adjustment. In our multivariable analysis, risk of infection remained significantly higher for occupational groups with frequent exposure to patients with covid-19 and those working in an emergency department or inpatient ward setting or working as a healthcare assistant. These findings underscore the importance of research into the nature of healthcare workers’ role and setting specific contact with patients and staff, even in the context of vaccination. Combined, our analyses highlight the crucial role of rapid covid-19 vaccine deployment in averting infections in our cohort during the second wave and the importance of equitable rollout throughout the healthcare workforce.

### Strengths and weaknesses of study

SIREN is a large prospective cohort study that is well positioned to explore the incidence of SARS-CoV-2 infection in the hospital workforce. We have well defined previous exposure history from an enrolment questionnaire (with negligible impact from missing data), a frequent PCR testing schedule, and laboratory records since the pandemic began through SGSS, regardless of participants’ enrolment date. This study gains considerable strength from our multifaceted approach, using both statistical and mathematical modelling built on data from SIREN and additional national datasets.

The main limitation of this study is the lack of detail at organisational level, which is needed to explore the impact of particular infection prevention and control policies during the second wave and organisational infrastructure. Disparities in infrastructure and pandemic response at the level of the organisation likely contributed to the residual variance seen between organisations that was not accounted for by our regression model. This study is also unable to unpick the behavioural nature of the demographic and occupational risk factors observed, which might contribute to the higher adjusted odds ratio of infection, but not unadjusted odds ratio, in male compared with female healthcare workers and the higher adjusted odds ratio in healthcare assistants, but not nurses, compared with office workers. Understanding the behavioural elements contributing to these findings is important in order to identify people at risk and target appropriate infection prevention and control measures accordingly. Clearly, frequent close proximity to patients with covid-19 is a strong risk factor, and the nature of an individual healthcare worker’s contact with patients and working practices is likely to modify this.^39^

A limitation of the mathematical modelling work included in this study is that we used empirical data to inform the community prevalence in non-healthcare workers: it does not incorporate a probable counterfactual increase in community cases in the absence of vaccines.^40^ Hence, in the simulation results, the contribution of community acquired infection in patient facing hospital healthcare workers to the overall infection risk in the “no vaccine” scenario is underestimated, and the simulated 9.9% reduction is a conservative figure.

### Strengths and weaknesses in relation to other studies

The Office for National Statistics’ population SARS-CoV-2 prevalence study reported infection in 4.2% (95% confidence interval 3.7% to 4.6%) of healthcare professionals between 1 September 2020 and 7 January 2021. Another study, REACT-1, reported infection in 2.1% (1.8% to 2.5%) and 0.7% (0.5% to 0.9%) of healthcare workers with direct patient contact between 6-22 January and 4-23 February, respectively.^41^ ^42^ ^43^ These findings should be compared with ours with caution: these studies consider different periods of the second wave, have less temporally dense testing protocols, have wider occupational definitions (for example, including primary care), and do not consider vaccination or susceptibility status. Our data are more relevant to hospital healthcare settings and better suited to guide hospital level policy.

Although studies before the vaccine rollout have previously highlighted demographic and occupational risk factors for SARS-CoV-2 infection,^13^ ^15^ ^44^ our study adds significantly to the literature by considering a period during rapid vaccination coverage, allowing the identification of risk factors that remain in the context of vaccination. Our chosen period of observation is likely the last opportunity in this pandemic to study a truly susceptible cohort and identify these demographic and occupational characteristics, as infection and vaccination patterns since the emergence of the omicron variant in November 2021 have led to very few people in the UK remaining fully susceptible. Future studies might struggle to recruit enough susceptible participants to have the statistical power needed to explore occupational risk factors for SARS-CoV-2, which are likely to contribute to future winter pressures.

### Meaning of study: possible explanations and implications for clinicians and policy makers

This study shows a clear effect of vaccines on the incidence of SARS-CoV-2 infection in SIREN participants: without the vaccine rollout, which prioritised frontline healthcare workers, staff absence due to covid-19 could have been 69% higher during the second wave, threatening healthcare provision. Patients’ safety could have been affected both directly, through an increase in downstream nosocomial infections, and indirectly, through staff shortages, if the vaccine rollout had not occurred when it did. The differential impact of vaccines on demographic and occupational groups can, in part, be explained by variations in speed of coverage; equitable opportunities for healthcare staff to be vaccinated must be prioritised, with monitoring of uptake and targeted encouragement in groups with lower uptake. This is particularly important during the emergence of variants of concern, such as the omicron variant, that may necessitate boosters for suitable protection.^26^

The occupational group with the strongest association with infection after adjustment was healthcare assistants (P<0.001), followed by bedside therapists (physiotherapists, occupational therapists, and speech and language therapists) (P=0.06), bringing into question whether the nature of their interaction with patients carries additional risk. The maintained risk in certain healthcare settings and roles after adjustment, likely due to residual confounders not included in our model, highlight the importance of understanding the nature of staff-patient contact and staff-staff mixing in different healthcare worker groups and settings, including households,^45^ and consideration of whether factors such as optimisation of personal protective equipment could reduce infection rates in these at-risk groups.^46^

### Unanswered questions and future research

This study reinforces the importance of vaccination among healthcare workers during a significant wave of the SARS-CoV-2 pandemic in England: mechanisms to ensure high vaccination coverage, including boosters, throughout the workforce must be used to minimise the impact of future waves on the NHS and healthcare provision. Focused patient and participant involvement work will be essential in understanding the barriers to achieving high vaccination coverage within at-risk demographic and occupational groups, including those impeding access and increasing hesitancy. This will be particularly important given the need for ongoing booster vaccination coverage to improve protection against the omicron variant, and the likely continued need for boosters for future variants of concern.

Future work should explore variability in the built environment, infection prevention and control and personal protective equipment policy, and ward based pressures across organisations and their associations with infection risk. Greater understanding of transmission dynamics among healthcare workers, particularly according to role and setting, will support NHS trusts in protecting their workforce and patients from SARS-CoV-2 infection and potentially other seasonal winter viruses.

## What is already known on this topic

Apparent and occupational risk factors for primary SARS-CoV-2 infection in healthcare workers were established before vaccine rollout, guiding local vaccine delivery
During rollout, coverage varied between healthcare worker groups, leading to disparities in exposure and protection across the workforce

## What this study adds

The rapid covid-19 vaccine rollout from December 2020 averted infection in a large proportion of hospital healthcare workers in England
Demographic and occupational risk factors persisted in healthcare workers despite vaccine rollout and should guide further infection prevention and control measures

## Data Availability

The metadata for this analysis will be available on reasonable request to researchers through the Health Data Research UK CO-CONNECT platform and available for secondary analysis.

## Web extra

Extra material supplied by authors

Web appendix: Appendix 1: Mathematical modelling methods

Web appendix: Appendix 2: Supplementary tables and figures

Web appendix: Appendix 3: SIREN Study Group members

## Notes

Contributors: DP, EJMM, and SE are joint first authors and contributed equally: order was decided by first name alphabetical order. CSB, JR, SH, and VJH conceived the study. AA, CSB, EJMM, EW, JI, SF, SH, VJH, and the SIREN Study Group delivered the SIREN study, including recruitment, data collection, and project management. DP and EJMM did the statistical analyses. DP, JR, RH, and SE contributed to and conducted the mathematical modelling. DP, EJMM, JI, SE, and VJH drafted the manuscript and supplementary materials. CSB, JR, SH, and VJH critically revised the manuscript for intellectual content. All authors read and approved the final manuscript. The corresponding author attests that all listed authors meet authorship criteria and that no others meeting the criteria have been omitted. SH is the guarantor.

The lead author (the manuscript’s guarantor) affirms that this manuscript is an honest, accurate, and transparent account of the study being reported; that no important aspects of the study have been omitted; and that any discrepancies from the study as planned (and, if relevant, registered) have been explained.

Dissemination to participants and related patient and public communities: The results of this study will be shared with participants and the network of SIREN study teams through regular participant and research site newsletters and webinars. Lay summaries will be produced and shared through social media and an upcoming website.

Provenance and peer review: Not commissioned; externally peer reviewed.
