# Supplementary material for: Burden of SARS-CoV-2 infection in healthcare workers during second wave in England and impact of vaccines: prospective multicentre cohort study (SIREN) and mathematical model
Source: BMJ. 2022 Jul 20;378:e070379. doi: 10.1136/bmj-2022-070379 (PMC9295077; doi:10.1136/bmj-2022-070379)
Supplement: Supplementary file 1 — Web appendix: Appendix 1: Mathematical modelling methods [file popd070379.ww1.pdf]

Appendix 1: Mathematical Modelling Methods

An individual-based model of the spread of COVID-19 in English hospitals:

Overview, Design Concepts, and Details (ODD) Protocol:
We have developed an individual-based model (IBM) of the transmission of COVID-19 in an English hospital and use the ODD protocol,[1] to provide a complete and rigorous model description of the model used for the simulations described in the "Mathematical Model" section of the main document's methods.

Purpose:
The purpose of the model is to represent the acquisition and transmission of SARS-CoV-2 by patient-facing healthcare workers (HCWs) and patients in a typical English hospital and answer questions around the source of infection and the effectiveness of interventions.

Entities, state variables and scales:
The model contains two environment classes, namely the Community and the Hospital. The Community class tracks the change in community prevalence per day and determines the number of community-acquired symptomatic and exposed cases to admit to hospital. The Hospital class stores the agent lists and handles the creation of newly admitted agents as well as the removal of patients that are to be discharged. There are two types of agents in the model, Patients, and HCWs, both of which inherit basic variables from the abstract People class.

Table 1: States and variables of model agents.

Variable	*Definition*
Community	
prevalence	Population prevalence at current time-step. Taken to be 1.5*admissions rate per bed (See **Error! Reference source not found.**).
infCases	Number of people currently infected. It is the product of population size and prevalence
expCases	Number of people exposed in community<br>*infCases** 1/(1-asymptomaticProb)
region	Region to be simulated from:<br>"North West", "North East and Yorkshire", "South West", "South East", "London", " Midlands", "East of England"
prop_alpha_comm/hosp	Proportion of cases in the community or hospital admissions that are with Alpha variant.
ld_alpha	Logistic distribution for proportion of community-acquired hospital cases that are Alpha variant.
ld_vacc_age_group	Logistic distribution for vaccination probability in new admissions. Age groups are: 16-49, 50-54,55-59, 60-64, 65-69,90-74, 75-79, 80+.
Hospital	
hospital	Array list containing *Parameters.wards* 'wards' that are also array lists, each of which contains *Parameters.shared_bays* 'bays' (also array lists) that contain *Parameters.bedsPerBay* 'beds', where each position in the beds array list either contains a single patient agent or is set to null.
red_wards	Array list of the position of those wards that contain known or suspected COVID-19 patients in the hospital ward array list
green_wards	Array list of the position of those wards that contain no known or suspected COVID-19 patients in the hospital ward array list
red_to_green_wards	Array list of the position of those wards to move from red to green at the

	end of the time-step
green_to_red_wards	Array list of the position of those wards to move from green to red at the end of the time-step
hcws_on_wards	Array list containing a separate array list for each ward that records which HCWs have visited that ward in a time-step
infectedPatientsByBay	Map of the number of patient agents in each bay that are infected
People	
id	Unique identifier for agents
Infection_Status	Enum for infection states. One of:<br>NONE (never infected),<br>EXPOSED (infected but not yet detectable or infectious. After a pre-defined incubation period these individuals transition to one of the infectious states),<br>INFECTED_P (pre-symptomatically infected, PCR-detectable and infectious but not yet symptomatic. These individuals become symptomatically infected after a pre-defined onset period has passed),<br>INFECTED_A (asymptomatically infected, PCR-detectable and infectious),<br>INFECTED_S (symptomatically infected, PCR-detectable and infectious),<br>RECOVERED (recovered/immune, non-infectious, assumed to be a permanent state).
Infection_status	Current infection status
recoveryStep	Time-step to recover from infection (if no infection then 1000000000)
incubationPeriod	Time-step to transition from exposed to pre-symptomatic (if no infection then 1000000000)
onsetPeriod	Time-step from pre-symptomatic to symptomatic (if no infection then 1000000000)
Type	Enum for type of person agent (Patient/HCW)
type	Type of this agent
Gender	Enum for gender. Male/Female
gender	Gender of person agent
communityInfection	Boolean for whether agents have a community acquired infection or not
timeInSim	Length of time agent has been in simulation
infected_by	Type and ID of agent that caused the infection (or Comm if infected in the community).
infection_type	Direct or indirect
vaccinated1	Boolean for whether person ever received a first dose of vaccine
vaccinated2	Boolean for whether person ever received a second dose of vaccine
infected	Boolean for whether person was ever infected
vaccinationDay1	Day that individual will become (HCWs) or became (patients) effectively vaccinated following a first dose of a vaccine
vaccinationDay2	Day that individual will become (HCWs) or became (patients) effectively vaccinated following a first dose of a vaccine
StrainCarried	Enum for possible strains to be carried (B1_1_7, OTHER}
strain_carried	Strain carried by infected individual
Patients	
Ward	Enum for ward type. One of: NEW_ADMISSION, GENERAL, SUSPECTED, TEST_NEG, TEST_POS, DISCH
dischargeTimeSteps	The time in steps that a patient will be discharged
dead	Boolean for live/dead patient
knownInfected	Boolean for identified infection
readmission	Boolean for whether patient has been readmitted or not

timeSinceDisch	Number of time-steps that the patient has been discharged for
carehome	Boolean for whether patient is from a care home or not
followOnDisch	Boolean for whether patient should be followed up on discharge or not.
tested	Flag for whether patient was tested again after 5-7 days (to ensure they only get tested once in this interval)
HCWs	
onShift	Boolean for whether HCW is currently on shift or at home
absentThroughSickness	Boolean for whether HCW is currently isolating
stepToReturn	Time-step to end self-isolation and return to work
shiftStartStep	Time-step that shift began or will begin if currently off shift
shiftEndStep	Time-step that shift ended or will end if currently on shift
tested	Boolean variable for whether HCW has been tested

Process overview and scheduling:
The model runs with a 4-hour time-step, i.e. six time-steps per day. Currently all agents are on the same time-step schedule and are handled by the agent scheduler built into the Multi-Agent Simulator of Neighbourhoods toolkit (MASON),[2] which randomly shuffles agents so that there is no order of which class/agent is stepped first, so the order of the processes active at every time-step described here is arbitrary.

Community: When a community object is instantiated, it randomly selects a region and reads in the admissions data for that region from the file "region_name.csv". Every time the community object is stepped it performs three basic tasks. It updates the prevalence variable in line with the admissions data that has been read in, calculated the number of infected cases in the community, and calculated the number of exposed cases in the community. The parameters for the logistic curves defining the proportion of hospital cases that are with the Alpha variant are different between regions. This distribution and the vaccination probability over time by five-year age bands are generated when the community object is instantiated.

Hospital: At the very first time-step the function *setupHospital* that reads in the number of HCWs and beds from the parameter file and creates all the HCW and patient agents required, each with *infection_status* = NONE, and randomly assigns each HCW's *onShift* variable. At each subsequent time-step the number of infected, susceptible, and exposed to admit from the community in this 24-hour period is calculated. The number of infected patients to admit is taken from the admissions file in the community object, and the *communityInfection* variable is set to true, then for a random number of the remaining empty beds patients are admitted as either susceptible or exposed. The exposure probability is proportional to the number of infected patients being admitted at that time (*Patients.expOnAdmissionProb* in the parameter file). Next the hospital's ward classifications are updated: making any red wards that have no known or suspected COVID-19 patients green and any wards with known or suspected COVID-19 patients red. Finally the list of HCWs that visit the ward in that time-step is reset ready for the next iteration of the hospital. On admission patients are assigned a length of stay (LOS) from a Weibull distribution that is a function of their age, gender, and infection status. The hospital is divided into *wards* "wards" with *shared_bays* "bays" in each ward and *bedsPerBay* beds in each bay (default *bedsPerBay* = 6). There are two locations for HCWs in a hospital instance, either in the hospital (can interact with all other HCWs in the hospital) or out of the hospital (implicitly interact with the community), and HCWs that are in the hospital interact with patients by randomly selecting beds within bays within wards to visit, during which time they interact with only the patient in that bed and no other patients or HCWs. Other spatial aspects of a hospital (wards, corridors, offices etc) are not explicitly represented. A subset of HCWs are defined to be *wardBased*, those HCWs only select a bed/bay to visit since their activities are restricted to a single ward defined on admission.

People: At each time-step every person agent which has *infection_status* = NONE calculates the probability of becoming exposed based on the number of infected patients they are exposed to on each ward, the number of infected HCWs they are exposed to in the hospital (randomly sampled from the number of infected HCWs and non-infected HCWs), and the transmission rates bP2P, bP2H, bH2H and bH2H from the parameter file. The transmission probabilities are assumed to be the absolute probability of transmission across all possible modes (airborne, aerosol, fomite) from each specified source. People that have *infection_status* = EXPOSED are assigned an incubation period from a Gamma distribution after which time they become asymptomatically or pre-symptomatically infected and are assigned a recovery time-step from a Gamma distribution. People with *infected_status* = INFECTED_P are assigned an onset period from a log-normal distribution after which time they transition to INFECTED_S. When the recovery time-step is exceeded, people have *infection_status* RECOVERED, and are immune to future infections (as reinfections were rare during this stage in the pandemic)[3, 4]. If the person agent is a Patient then when they become infected a new LOS is generated. Currently there is no rule stating that this should be greater than the original LOS. Note that the recovery time-step can be greater than the LOS.

Patients: As well as undergoing the behaviours that apply to all People agents in the hospital, patients can be tested on admission and are assigned to either single rooms or multiple bed bays, selected following the flow diagram in **Error! Reference source not found.**. They are also discharged and those that have an *infection_status* of EXPOSED or INFECTED_P on admission are stepped for a further 14 days. During this time, the same Patient agent may be readmitted to hospital. On admission patients may have been vaccinated with one or two doses of a vaccine, with the vaccination probability being drawn from the dose-specific logistic distribution for their age group from the Community class. We took vaccination data from National Immunisation Management System (NIMS), with population denominators taken from Office for National Statistics (ONS) population predictions for 2020,[5] stratified for age-band included in the Joint Committee on Vaccination and Immunisation (JCVI) priority list.[6] It is assumed that no COVID-19 admissions are vaccinated at this stage. Patients that share a bay with an infected patient can be infected directly with transmission probability *bP2P* and all patients in the ward can become infected indirectly by any other infected patient based on the number of infected patients in the hospital with probability *P2P_hosp*. Patients can also be directly or indirectly infected by any infected HCW that is on shift with probability *bH2P,* or indirectly by a patient where there is shared HCW contacts again with probability *bP2P_hosp*. We have calibrated these probabilities to reproduce observed data on the proportion of inpatients infected at an individual secondary healthcare organisation (Trust) level.

HCW: In addition to the behaviours exhibited by all People agents, HCWs iterate between on and off shift, where they are infected by either other people in the hospital (if *onShift* = true) or with a probability of 0.13*beta*community prevalence per day if *onShift* = false . This is derived from data stating that each case has on average seven contacts. If 1.4 million/66.4 million people work for the NHS,[7] that means that ~2% of people in England are classed as HCWs in this setting, therefore the probability of one of these seven contacts being a HCW is 1 – (probability none of them are) = 1-0.98^7 = 0.136. For each contact the transmission rate is approximately 0.4 thus the probability of a HCW picking up an infection in the community is 0.136*0.4*community incidence rate (prevalence/4.6 days) where 0.4 is the transmission rate in the community.[8] HCW agents can also self-isolate while their *infection_status* is INFECTED_S, they can also be periodically tested with lateral flow device (LFD) tests and those with positive results are forced to isolate, with two days isolation following a false positive or ten days isolation following a true positive. HCWs that have been isolating following a true positive are automatically classed as RECOVERED when returning to work. The time that a HCW will become vaccinated (for first and second doses) is assigned on

instantiation and is selected from dose-specific logistic distribution, we calculated the distribution function parameters from SIREN study participants linked with NIMS.

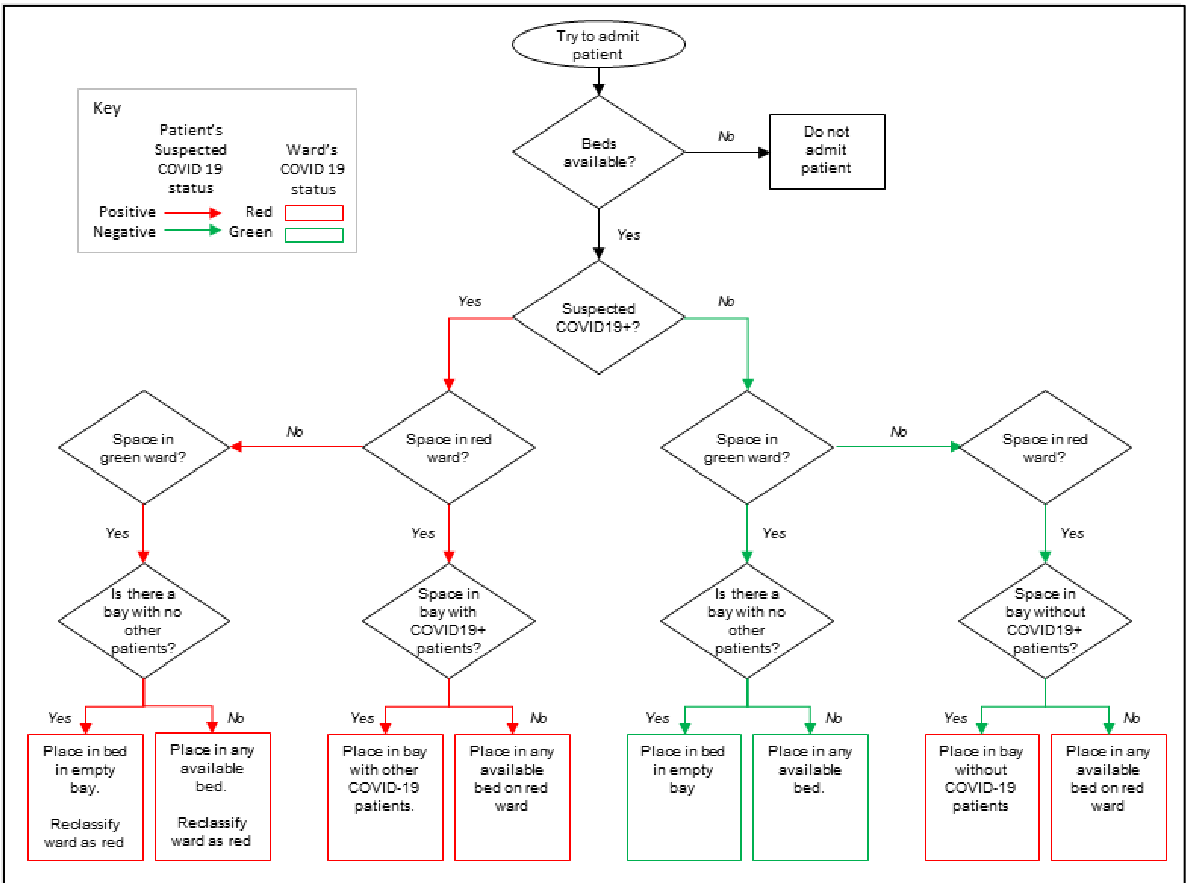

Figure 1: Schematic of assignment of patients to beds.

Design Concepts:
The model is designed with the structure demonstrated in Additional Figure 3 in the Supplementary Information: Additional Tables and Figures.

The emergent properties of this model are number of infected patients and HCWs over time and the sources of infection. Parameter uncertainty is introduced by using probabilities for infection rates and the inclusion of LOS, incubation, onset, recovery, and vaccination variables drawn from probability distributions.

We parameterised this model for a typical English hospital with 1000 beds and 8000 HCWs to reflect the average bed size to staff ratio in NHS England (NHSE) Trusts. We estimated death rates and length of stay distributions using the R library *fitdistrplus*,[9] and data from the Secondary Uses Service (SUS) COVID-19 linked to laboratory data of positive tests from Public Health England's Second Generation Surveillance System (SGSS) (methodology as in Bhattacharya et al.).[10] The death probability was estimated by fitting a polynomial to the average probability of dying in hospital per year of age multiplied by the discharge probability from the SUS dataset, sampling the age distribution from the SUS dataset, and taking the mean. For susceptible individuals, the death probability was calculated from the literature,[11] and for infected individuals, the combined SUS/SGSS data were used. Data gathered from SUS comprised all completed hospital spells in NHSE Trusts arising from admissions over a nine-month period from 10 March 2020 through to 31 December 2020 inclusive. We obtained age (in years) and sex of the patient, and calculated length of stay to

the nearest day using recorded admission and discharge dates. We fitted length of stay distributions for positive and negative admissions using a Weibull distribution and adjusting for age and sex. Suitable values for transmission parameters between patients and HCWs were selected to quantitively and qualitatively reproduce data from individual Trusts in different regions of England, with differential case-loads. We parameterised vaccine effectiveness using data relating to the protection against infection by Alpha variant (as the predominate variant across the vaccine rollout period within the second wave) by Pfizer BioNTech (BNT162b2) COVID-19 vaccine (received by over 90% of vaccinated SIREN participants) which was assumed constant across the working-age range.[12] HCW infection data was calibrated using a meta-analysis of studies on HCW infection rates,[13] combined with results from the SIREN study.

Model Calibration:
Where data were available, we assigned values to identifiable parameters from the literature (

Appendix Table A*1*). The unidentifiable parameters were iteratively calibrated to within acceptable ranges using the methodology described by Joslyn et al.[14] We derived a set of criteria against which simulations were deemed to be representative of observed data using aggregated hospital-level data on the infection rate in patients and HCWs drawn; these criteria are defined in Table 2. Initially log-10 uniform probability distributions were assumed for all transmission probabilities and parameters were allowed to take on any value from 10^{-10} to 1. 1000 Latin Hypercube sampled parameter sets were generated and simulations were executed as described in "Simulations". For the next iteration a new distribution was derived for each parameter set by identifying the highest-density regions for each parameter set in which any of the tests were satisfied. This was repeated for two further iterations until a subset of runs passed all four tests. The parameter sets that passed all four tests were then assessed qualitatively against empirical data on patient and HCW infections, so compared qualitatively to the parameter values to those in a previously published simple deterministic model of hospital transmission.[15]

The proportion of susceptible patients that acquired a nosocomial infection was estimated to be less than 5% based on a previous modelling study demonstrating this outcome in a high prevalence area with higher than normal transmission rates.[15] We used data from the SIREN study to estimate the proportion of HCWs infected over time. The range into which a result must fall to pass the test relating to each criterion is detailed in Table 2.

Table 2: Criteria for an outcome to be defined as related to a feasible parameter set.

Criteria		Min	Max
1	Maximum proportion of susceptible patients that develop a nosocomial infection	0.0005	0.05
2	Proportion of HCWs infected before 10 November 2020	0.202	0.353
3	Proportion of HCWs infected before 08 December 2020	0.208	0.382
4	Proportion of HCWs infected between 10 November and 08 December 2020	0.003	0.021

Supplementary Information: Additional Tables and Figures, Additional Figure 3 shows the distribution of cumulative infection in SIREN participants by Trust at the calibration timepoints.

Input Data:
The model relies on input data in the form of a file containing the number of cases to admit per day, this file must be named "region.csv" where region is one of those defined in Table 1. The parameters are also imported from an XML parameter file, examples of these files are contained in the Appendix below.

Computational platform:

The model is constructed in the Java programming language using Java SE 11. The IBM is cross-platform and runs without visualisation software. Simulations were executed locally.

Simulations:
The model was simulated for 2550 time-steps covering a time period of 425 days (6 steps per simulated day) aligning with the collection period of the patient-level and SIREN data. Individual-level patient and HCW data on infection status and location were recorded at each time-step.

Scaling between regional and national data:
To scale regional data up to a national level, the regional data is weighted by the proportion of all NHS beds in a particular region.[16]

References:

1. Grimm V, Berger U, DeAngelis DL, et al. The ODD protocol: A review and first update. *Ecological Modelling* 2010;221(23):2760-68. doi: doi.org/10.1016/j.ecolmodel.2010.08.019.
2. Luke S, Cioffi-Revilla C, Panait L, Sullivan K, Balan G. MASON: A multiagent simulation environment. *Simulation* 2005;81(7):517-27. doi: 10.1177/0037549705058073.
3. Hanrath AT, Payne BAI, Duncan CJA. Prior SARS-CoV-2 infection is associated with protection against symptomatic reinfection. *J Infect* 2021;82(4):e29-e30. doi: 10.1016/j.jinf.2020.12.023.
4. Hall VJ, Foulkes S, Charlett A, et al. SARS-CoV-2 infection rates of antibody-positive compared with antibody-negative health-care workers in England: a large, multicentre, prospective cohort study (SIREN). *Lancet* 2021;397(10283):1459-69. doi: 10.1016/s0140-6736(21)00675-9.
5. Office for National Statistics. National population projections: 2018-based. Office for National Statistics. https://www.ons.gov.uk/peoplepopulationandcommunity/populationandmigration/populationprojections/bulletins/nationalpopulationprojections/2018based.
6. Joint Committee on Vaccination and Immunisation. Joint Committee on Vaccination and Immunisation: advice on priority groups for COVID-19 vaccination, 30 December 2020. 6 January 2021. https://www.gov.uk/government/publications/priority-groups-for-coronavirus-covid-19-vaccination-advice-from-the-jcvi-30-december-2020/joint-committee-on-vaccination-and-immunisation-advice-on-priority-groups-for-covid-19-vaccination-30-december-2020.
7. The Kings Fund. Overview of the health and social care workforce. https://www.kingsfund.org.uk/projects/time-think-differently/trends-workforce-overview.
8. Birrell P, Blake J, Van Leeuwen E, Gent N, De Angelis D. Real-time nowcasting and forecasting of COVID-19 dynamics in England: the first wave. *Philos Trans R Soc Lond B Biol Sci* 2021;376(1829):20200279. doi: 10.1098/rstb.2020.0279.
9. Delignette-Muller ML, Dutang CJJoss. fitdistrplus: An R package for fitting distributions. 2015;64(4):1-34. doi: 10.18637/jss.v064.i04.
10. Bhattacharya A, Collin SM, Stimson J, et al. Healthcare-associated COVID-19 in England: a national data linkage study. *J Infect* 2021 doi: 10.1016/j.jinf.2021.08.039.
11. Cohen J, Bilsen J, Addington-Hall J, et al. Population-based study of dying in hospital in six European countries. *Palliat Med* 2008;22(6):702-10. doi: 10.1177/0269216308092285.
12. Hall VJ, Foulkes S, Saei A, et al. COVID-19 vaccine coverage in health-care workers in England and effectiveness of BNT162b2 mRNA vaccine against infection (SIREN): a prospective, multicentre, cohort study. *Lancet* 2021;397(10286):1725-35. doi: 10.1016/s0140-6736(21)00790-x.
13. Evans S, Stimson J, Pople D, et al. Quantifying the contribution of pathways of nosocomial acquisition of COVID-19 in English hospitals. *Int J Epidemiol* 2021 doi: 10.1093/ije/dyab241.
14. Joslyn LR, Kirschner DE, Linderman JJ. CaliPro: A Calibration Protocol That Utilizes Parameter Density Estimation to Explore Parameter Space and Calibrate Complex Biological Models. *Cell Mol Bioeng* 2021;14(1):31-47. doi: 10.1007/s12195-020-00650-z.
15. Evans S, Agnew E, Vynnycky E, et al. The impact of testing and infection prevention and control strategies on within-hospital transmission dynamics of COVID-19 in English hospitals. *Philos Trans R Soc Lond B Biol Sci* 2021;376(1829):20200268. doi: 10.1098/rstb.2020.0268.
16. NHS England. Bed Availability and Occupancy Data – Overnight. https://www.england.nhs.uk/statistics/statistical-work-areas/bed-availability-and-occupancy/bed-data-overnight/.
17. Nuffield Trust. Hospital bed occupancy. https://www.nuffieldtrust.org.uk/resource/hospital-bed-occupancy.

18. Woloshin S, Patel N, Kesselheim AS. False Negative Tests for SARS-CoV-2 Infection - Challenges and Implications. *N Engl J Med* 2020;383(6):e38. doi: 10.1056/NEJMp2015897.
19. University of Oxford SARS-CoV-2 test development validation cell. Preliminary report from the Joint PHE Porton Down & University of Oxford SARS-CoV-2 test development and validation cell: Rapid evaluation of Lateral Flow Viral Antigen detection devices (LFDs) for mass community testing. https://www.ox.ac.uk/sites/files/oxford/media_wysiwyg/UK%20evaluation_PHE%20Porton%20Down%20%20University%20of%20Oxford_final.pdf.
20. He X, Lau EH, Wu P, et al. Temporal dynamics in viral shedding and transmissibility of COVID-19. *Nature medicine* 2020;26(5):672-75. doi: 1038/s41591-020-0869-5.
21. Office for National Statistics. Population estimates for the UK, England and Wales, Scotland and Northern Ireland: mid-2018. Office for National Statistics; 27 June 2019. https://www.ons.gov.uk/peoplepopulationandcommunity/populationandmigration/populationestimates/bulletins/annualmidyearpopulationestimates/mid2018.
22. Office for Health Improvement Disparities. AMR local indicators - OHID. https://fingertips.phe.org.uk/amr-local-indicators#gid/1938132917/ati/118.
23. Cohen B, Hyman S, Rosenberg L, Larson E. Frequency of patient contact with health care personnel and visitors: implications for infection prevention. *The Joint Commission Journal on Quality and Patient Safety* 2012;38(12):560-65. doi: 10.1016/s1553-7250(12)38073-2.
24. Public Health England. UK flu levels according to PHE statistics: 2018 to 2019. https://www.gov.uk/government/news/uk-flu-levels-according-to-phe-statistics-2018-to-2019.
25. Kluytmans-van den Bergh MFQ, Buiting AGM, Pas SD, et al. Prevalence and Clinical Presentation of Health Care Workers With Symptoms of Coronavirus Disease 2019 in 2 Dutch Hospitals During an Early Phase of the Pandemic. *Jama Netw Open* 2020(2574-3805 (Electronic)) doi: doi: 10.1001/jamanetworkopen.2020.9673.
26. NHS England, NHS Improvement. Novel coronavirus (COVID-19) standard operating procedure NHS England and NHS Improvement rollout of lateral flow devices for asymptomatic staff testing for SARS CoV-2 (phase 2: trusts). 16 November 2020 https://www.england.nhs.uk/coronavirus/wp-content/uploads/sites/52/2020/11/C0873_i_SOP_LFD-rollout-for-asymptomatic-staff-testing_phase-2-trusts-v1.1_16-nov20.pdf.
27. Jarvis C, Edmunds J, CMMID COVID-19 Working Group. Social contacts in workplace the UK from the CoMix social contact survey. 29 June 2021. https://assets.publishing.service.gov.uk/government/uploads/system/uploads/attachment_data/file/1009160/S1311_Comix_Report_contacts_in_the_workplace_SAGE_93.pdf.
28. Harris RJ, Hall JA, Zaidi A, et al. Effect of Vaccination on Household Transmission of SARS-CoV-2 in England. *N Engl J Med* 2021;385(8):759-60. doi: 10.1056/NEJMc2107717.
29. Andrews N, Tessier E, Stowe J, et al. Vaccine effectiveness and duration of protection of Comirnaty, Vaxzevria and Spikevax against mild and severe COVID-19 in the UK. *Medrxiv* 2021. doi: 10.1101/2021.09.15.21263583.
30. Volz E, Mishra S, Chand M, et al. Assessing transmissibility of SARS-CoV-2 lineage B.1.1.7 in England. *Nature* 2021;593(7858):266-69. doi: 10.1038/s41586-021-03470-x.

Appendices: Parameter Value Tables and Example Input Files:

Appendix Table A1: Parameter values.

General			
occupancy	Proportion of occupied beds in trust	0.85	Mean from individual trust data in SUS
hcws	Number of HCWs	8000	Mean from individual trust data
patients	Number of patients initially in trust	860	At 86% capacity[17]
testSens_PCR	Sensitivity of PCR test (false negative rate)	0.95	Woloshin et al.[18]
testSpec_PCR	Specificity of PCR test (false positive rate)	0.995	Woloshin et al.[18]
testSens_LFD	Sensitivity of LFD test (false negative rate)	0.78	PHE/University of Oxford[19]
testSpec_LFD	Specificity of LFD test (false positive rate)	0.995	PHE/University of Oxford[19]
incubationPeriodDays_shape	Shape variable for Gamma distribution for incubation period	13.3	He et al.[20]
incubationPeriodDays_rate	Rate variable for Gamma distribution for incubation period	4.16	He et al.[20]
onsetPeriodDays_mean	Mean for Log-normal distribution for onset period	1.434065	SPI-M communication
onsetPeriodDays_sd	SD for Log-normal distribution for onset period	0.6612	SPI-M communication
camPop	Community population (Cambridgeshire)	651482	2018 mid-year estimate[21]
wards	Number of wards	42	
shared_bays	Number of bays	4	~80% beds are in shared rooms[22]
bedsPerBay	Number of beds in a shared room	6	
Patients			
probMale	Probability of patients being Male	0.576	SUS
ageShape	Shape component of Gamma distribution of age distribution	4.145	Fit to data from SUS
ageScale	Scale component of Gamma distribution of age distribution	75.48	Fit to data from SUS
shapeLOS_Susc	Shape parameter for LOS distribution Weibull distribution for susceptible patients	-0.1780082	Fit to data from SUS
scaleLOS_Susc_int	Intercept for scale parameter for LOS distribution Weibull distribution for susceptible patients	2.331949	Fit to data from SUS
scaleLOS_Susc_age	Age component of scale parameter for LOS distribution Weibull distribution for susceptible patients	-0.00546131	Fit to data from SUS
scaleLOS_Susc_female	Female gender component of scale parameter for LOS distribution Weibull distribution for susceptible patients	-1.242609	Fit to data from SUS
scaleLOS_Susc_age_female	Interaction of age and female gender component of scale parameter for LOS distribution Weibull distribution for	0.01424664	Fit to data from SUS

	susceptible patients		
shapeLOS_Inf	Shape parameter for LOS distribution Weibull distribution for symptomatically infected patients	0.07853536	Fit to data from SUS
scaleLOS_Inf_int	Intercept for scale parameter for LOS distribution Weibull distribution for symptomatically infected patients	2.595361	Fit to data from SUS
scaleLOS_Inf_age	Age component of scale parameter for LOS distribution Weibull distribution for susceptible patients	0.002301093	Fit to data from SUS
scaleLOS_Inf_female	Female gender component of scale parameter for LOS distribution Weibull distribution for susceptible patients	-0.5465226	Fit to data from SUS
scaleLOS_Inf_age_female	Interaction of age and female gender component of scale parameter for LOS distribution Weibull distribution for susceptible patients	0.006978023	Fit to data from SUS
patientAsymProb	Probability a patient infection is asymptomatic	0.4	SUS
bP2P	Transmission rate to other patients		Calibrated
bH2P	Transmission rate from HCW to patients		Calibrated
bP2P_hosp	Indirect transmission rate from patients to other patients		Calibrated
recoveryRatePat_Shape	Shape parameter of Gamma distribution of recovery distribution per day.	1.43	Fit to data from SUS
recoveryRatePat_Rate	Rate parameter of Gamma distribution of recovery distribution per day.	0.542	Fit to data from SUS
deathOnDisch_Inf_Int	Intercept of linear model for probability of death on discharge for infected patients	-0.00043572	Fit to data from SUS
deathOnDisch_Inf_X	X component of linear model for probability of death on discharge for infected patients	-0.00159802	Fit to data from SUS
deathOnDisch_Inf_X2	X^2 component of linear model for probability of death on discharge for infected patients	7.30529E-05	Fit to data from SUS
deathOnDisch_Susc_Int	Intercept of linear model for probability of death on discharge for non-symptomatic/uninfected patients	-7.96613	Fit to data from Cohen et al.[11]
deathOnDisch_Susc_X	X component of linear model for probability of death on discharge for non-symptomatic/uninfected patients	0.00102	Fit to data from Cohen et al.[11]
deathOnDisch_Susc_X2	X^2 component of linear model for probability of death on discharge for non-symptomatic/uninfected patients	0.000896	Fit to data from Cohen et al.[11]
testOnAdmProb_Inf	Probability a symptomatically infected patient will be tested on	0.95	Estimated from individual NHSE trust

	admission		data
testOnAdmProb_Other	Probability non-infected patient will be tested at random on admission	0.05	Estimated from individual NHSE trust data
testInHospProb	Probability a symptomatically infected patient infected nosocomially will be tested per time-step	0.1	Assumption that all patients will be tested within 2 days of developing symptoms
testPeriodSteps	Time-steps from test to result	8	Estimated from individual NHSE trust data
readmitProb	Probability a patient that develops symptoms after discharge will be readmitted within 14 days	0.2	Estimated from individual NHSE trust data
expOnAdmissionProb	Probability a susceptible patient will be exposed on admission (to be multiplied by the number of cases admitted that are known symptomatic)	0.002631579	
retestProb_per_step	Probability a patient will be retested after day 5 (per step)	0	No retesting in this analysis
hcw_to_patient_contacts	Number of unique patients seen by a HCW every day (used to calculate exposure risk to COVID+ patients)	20	Cohen et al.[23]
non_covid_sympt_prob	Probability a non-infected patient with have COVID-19-like symptoms on admission	0.1	Assumption based on ILI proportions in a normal year[24]
HCWs			
shiftLengthHrs	Length of shift in hours (this is converted to time-steps within the model code)	12	Assumption
hcwAsymProb	Probability a HCW that becomes infected is asymptomatic	0.4	Assumption
bP2H	Transmission probability from patients to HCWs per time-step	0.0000025	Calibrated
bH2H	Transmission probability from HCWs to other HCWs per time-step	0.0000001	Calibrated
absentThroughSick_self	Probability a HCW will self-isolate per time-step	0.01	34% over total infected time[25]
testProbHCWDays	Periodicity of HCW LFD testing (days)	3.5	NHSE LFD protocol[26]
lfd_test_compliance	Proportion of HCWs adhering to LFD testing regime	0.7	Expert opinion
absentDays	Number of days to be off work following a positive test	7	
beginTestingDay	Day that LFD testing of HCW began	253	
commScale	Scale of community acquisition rate for HCWs	0.052	CoMIX[27]
patient_to_hcw_contacts	Number of HCWs seen by a patient per day (for scaling transmission risk)	18	Cohen et al.[23]

prob_ward_based	Probability a HCW is based on a ward instead of moving around the hospital	0.25	Estimate from proportion of nursing staff out of all staff
days_to_vaccine_efficacy	Day after vaccination that protection begins	21	
hcw_vaccination_start_day	Day that vaccination program in HCWs commences	281	8 December 2021
Comm			
careHomeProb	Probability a patient is from a care home	0.02	Health foundation communication
caseScale	Scaling factor for admissions rate	1	
vacc_scale_from	Scaling factor for transmissibility from vaccinated individual	0.5	Harris et al.[28]
vacc_scale_to1	Scaling factor for transmissibility to individual vaccinated with one dose (trans = 1-value)	0.7	Hall et al.[12]
vacc_scale_to2	Scaling factor for transmissibility to individual vaccinated with two doses (trans = 1-value)	0.7	Andrews et al.[29]
var_scale_alpha	Scaling factor of beta parameter for Alpha variant	1.56	

Appendix Table A2: Logistic distribution coefficients for Alpha variant proportion.

Fit to period 1st October – 22nd January inclusive (day 0 = 1 March 2020)
Fitted function : $y = A/(1 + exp(-k(t - \mu)))$
t = day number, y = coverage proportion

	Fitted coefficients			Observed data
NHSE Region	A	k	μ	Source
London	0.977	.0999	63.525	Volz et al.[30]
Midlands	0.907	0.1213	83.510	
North East and Yorkshire	0.842	0.1221	88.627	
North West	0.911	0.1071	89.180	
South East	0.915	0.0983	52.360	
South West	0.957	0.0871	81.662	
East of England	0.951	0.1019	65.037	

Fitted using R package ‘drc’

Appendix Table A3: Logistic distribution coefficients for vaccine cover proportion (1+ doses).

Fit to period 7th December – 9th April inclusive, assuming day 0 (7th December) has 0% coverage
Fitted function : $y = A/(1 + exp(-k(t - \mu)))$
t = day number, y = coverage proportion

		Fitted coefficients			Observed data
		A	k	μ	Source
HCW	England	0.931251619	0.116446417	31.90180448	SIREN-NIMS
Population	16-49	0.361368	0.0439	92.47233	NIMS, ONS[5]
	50-54	1.643953	0.049632	114.9436	
	55-59	1.257394	0.058007	99.44187	
	60-64	1.011423	0.089646	82.83167	
	65-69	1.011423	0.089646	82.83167	
	70-74	0.954113	0.227902	57.10629	
	75-79	0.979244	0.204977	48.48741	
	80plus	0.955866	0.109699	35.95469	

Input File Example 1: XML parameter file.

```
<?xml version="1.0" encoding="UTF-8" standalone="no"?>
<input>
    <General>
        <occupancy>0.85</occupancy>
        <doctors>1600</doctors>
        <nurses>6400</nurses>
        <hcws>8000</hcws>
        <patients>200</patients>
        <recruitPatInt>-5.062</recruitPatInt>
        <recruitPatX> 1.478</recruitPatX>
        <recruitPatX2>-0.03795</recruitPatX2>
        <recruitPatX3>0.0002646</recruitPatX3>
        <testSens>0.95</testSens>
        <testSpec>0.95</testSpec>
        <incubationPeriodDays_shape>13.3</incubationPeriodDays_shape>
        <incubationPeriodDays_rate>4.16</incubationPeriodDays_rate>
        <onsetPeriodDays_mean>1.434065</onsetPeriodDays_mean>
        <onsetPeriodDays_sd>0.6612</onsetPeriodDays_sd>
        <camPop>651482</camPop>
        <recScale>0.430980337293819</recScale>
        <asympCommScale>233.039498227648</asympCommScale>
```

```
        <wards>42</wards>
        <single_bays>0</single_bays>
        <shared_bays>4</shared_bays>
        <bedsPerBay>6</bedsPerBay>
    </General>
    <Patients>
        <probMale>0.576</probMale>
        <ageShape>4.145</ageShape>
        <ageScale>75.48</ageScale>
        <shapeLOS_Susc>-0.17800824</shapeLOS_Susc>
        <scaleLOS_Susc_int>2.33194900</scaleLOS_Susc_int>
        <scaleLOS_Susc_age>-0.00546131</scaleLOS_Susc_age>
        <scaleLOS_Susc_female>-1.24260868</scaleLOS_Susc_female>
        <scaleLOS_Susc_age_female>0.01424664</scaleLOS_Susc_age_female>
        <shapeLOS_Inf>0.078535365</shapeLOS_Inf>
        <scaleLOS_Inf_int>2.595360634</scaleLOS_Inf_int>
        <scaleLOS_Inf_age>0.002301093</scaleLOS_Inf_age>
        <scaleLOS_Inf_female>-0.546522561</scaleLOS_Inf_female>
        <scaleLOS_Inf_age_female>0.006978023</scaleLOS_Inf_age_female>
        <patientAsymProb>0.4</patientAsymProb>
        <bP2P>0.0022</bP2P>
        <bP2P_hosp>0.000015</bP2P_hosp>
        <bH2P>5.00E-04</bH2P>
        <recoveryRatePat_Shape>1.43</recoveryRatePat_Shape>
        <recoveryRatePat_Rate>0.542</recoveryRatePat_Rate>
        <deathOnDisch_Inf_Int>-0.0004357198</deathOnDisch_Inf_Int>
        <deathOnDisch_Inf_X>-0.0015980156</deathOnDisch_Inf_X>
        <deathOnDisch_Inf_X2>0.0000730529</deathOnDisch_Inf_X2>
        <deathOnDisch_Susc_Int>-7.96613</deathOnDisch_Susc_Int>
        <deathOnDisch_Susc_X>0.00102</deathOnDisch_Susc_X>
        <deathOnDisch_Susc_X2>0.000896</deathOnDisch_Susc_X2>
        <testOnAdmProb_Inf>1</testOnAdmProb_Inf>
        <testOnAdmProb_Other>1</testOnAdmProb_Other>
        <testInHospProb>0.2</testInHospProb>
        <testPeriodSteps>8</testPeriodSteps>
        <readmitProb>0.2</readmitProb>
        <expOnAdmissionProb>0.002631579</expOnAdmissionProb>
        <testPosScale>0.0</testPosScale>
        <testNegScale>1.0</testNegScale>
        <suspectedScale>1.0</suspectedScale>
        <retestProb_per_step>0.0</retestProb_per_step>
        <dayToRetest>10000</dayToRetest>
        <non_covid_sympt_prob>0.1</non_covid_sympt_prob>
        <hcw_to_patient_contacts>20</hcw_to_patient_contacts>
    </Patients>
    <HCWs>
        <shiftLengthHrs>12</shiftLengthHrs>
        <hcwAsymProb>0.4</hcwAsymProb>
        <bP2H>2.5E-04</bP2H>
        <bH2H>1.5E-04</bH2H>
        <absentThroughSick_self>0.1</absentThroughSick_self>
        <absentThroughSick_random>0.005</absentThroughSick_random>
        <testProbHCWDays>5000</testProbHCWDays>
        <absentDays>10</absentDays>
        <beginTestingDay>1</beginTestingDay>
        <commScale>0.2</commScale>
        <patient_to_hcw_contacts>18</patient_to_hcw_contacts>
        <prob_ward_based>0.25</prob_ward_based>
            <hcw_vaccination_start_day>281</hcw_vaccination_start_day>
            <days_to_vaccine_efficacy>21</days_to_vaccine_efficacy>
            <hcw_vaccine_eq_A>0.93</hcw_vaccine_eq_A>
```

```
            <hcw_vaccine_eq_k>0.116446417</hcw_vaccine_eq_k>
            <hcw_vaccine_eq_mu>31.90180448</hcw_vaccine_eq_mu>
            <vaccine_efficacy_one_dose>0.76</vaccine_efficacy_one_dose>
    </HCWs>
    <Comm>
        <commProbInt>-0.00000469835</commProbInt>
        <commProbX> 0.000001895326</commProbX>
        <commProbX2>-0.00000002551533</commProbX2>
        <minCommProb>0.0000015</minCommProb>
        <symptomToHospDays>0</symptomToHospDays>
        <careHomeProb>0.1</careHomeProb>
        <caseScale>1</caseScale>
        <visitorsPerStep>0</visitorsPerStep>
        <extraLOS>1.42</extraLOS>
            <prop_117_eq_A>1.0</prop_117_eq_A>
            <prop_117_eq_k>0.63</prop_117_eq_k>
            <prop_117_eq_mu>57.4</prop_117_eq_mu>
            <vacc_scale_from>0.5</vacc_scale_from>
            <var_scale_from>1.57</var_scale_from>
            <comm_vac_16_to_49_A>0.361</comm_vac_16_to_49_A>
            <comm_vac_50_to_54_A>1.643</comm_vac_50_to_54_A>
            <comm_vac_55_to_59_A>1.257</comm_vac_55_to_59_A>
            <comm_vac_60_to_64_A>1.011</comm_vac_60_to_64_A>
            <comm_vac_65_to_69_A>0.928</comm_vac_65_to_69_A>
            <comm_vac_70_to_74_A>0.954</comm_vac_70_to_74_A>
            <comm_vac_75_to_79_A>0.979</comm_vac_75_to_79_A>
            <comm_vac_80_plus_A>0.955</comm_vac_80_plus_A>
            <comm_vac_16_to_49_k>0.043</comm_vac_16_to_49_k>
            <comm_vac_50_to_54_k>0.049</comm_vac_50_to_54_k>
            <comm_vac_55_to_59_k>0.058</comm_vac_55_to_59_k>
            <comm_vac_60_to_64_k>0.089</comm_vac_60_to_64_k>
            <comm_vac_65_to_69_k>0.181</comm_vac_65_to_69_k>
            <comm_vac_70_to_74_k>0.227</comm_vac_70_to_74_k>
            <comm_vac_75_to_79_k>0.204</comm_vac_75_to_79_k>
            <comm_vac_80_plus_k>0.109</comm_vac_80_plus_k>
            <comm_vac_16_to_49_mu>92.472</comm_vac_16_to_49_mu>
            <comm_vac_50_to_54_mu>114.943</comm_vac_50_to_54_mu>
            <comm_vac_55_to_59_mu>99.441</comm_vac_55_to_59_mu>
            <comm_vac_60_to_64_mu>82.831</comm_vac_60_to_64_mu>
            <comm_vac_65_to_69_mu>68.039</comm_vac_65_to_69_mu>
            <comm_vac_70_to_74_mu>57.106</comm_vac_70_to_74_mu>
            <comm_vac_75_to_79_mu>48.487</comm_vac_75_to_79_mu>
            <comm_vac_80_plus_mu>35.954</comm_vac_80_plus_mu>
    </Comm>
</input>
```

Input File Example 2: Admissions File.

X	y	region
0	0	East Of England
1	0	East Of England
2	0	East Of England
3	0	East Of England
4	0	East Of England
5	0	East Of England
6	0	East Of England
7	0	East Of England
8	0	East Of England
9	0	East Of England
10	0.000336	East Of England
11	0.001532	East Of England
12	0.002727	East Of England
13	0.003923	East Of England
14	0.005119	East Of England
15	0.006315	East Of England
16	0.007508	East Of England
17	0.008691	East Of England
18	0.009854	East Of England
19	0.010982	East Of England
20	0.012061	East Of England
