# Supplementary material for: Burden of SARS-CoV-2 infection in healthcare workers during second wave in England and impact of vaccines: prospective multicentre cohort study (SIREN) and mathematical model
Source: BMJ. 2022 Jul 20;378:e070379. doi: 10.1136/bmj-2022-070379 (PMC9295077; doi:10.1136/bmj-2022-070379)
Supplement: Supplementary file 2 — Web appendix: Appendix 2: Supplementary tables and figures [file popd070379.ww2.pdf]

Appendix 2: Supplementary Tables and Figures.

Supplementary Table A: Page: 2
Crude attack rates for susceptible participants from devolved administrations, (with participants from England for comparison).

Supplementary Table B: Page: 3
Crude attack rates for subgroups excluded from the risk factor (regression) analysis due to missing characteristic data.

Supplementary Figure A: Page: 4-5
Weekly incidence and vaccine coverage in susceptible SIREN participants, compared with total incidence stratified by variant, by region.

Supplementary Figure B: Page: 6
Weekly incidence in susceptible SIREN participants, hospital patients (acute trusts), and non-hospitalised cases.

Supplementary Figure C: Page: 7-8
Mathematical model schematic, and dynamics for Alpha variant cases proportion and vaccination coverage

Supplementary Figure D: Page: 9
Cumulative infection in HCW across the pandemic with comparison of mathematical model (IBM) output and SIREN data.

Supplementary Figure E: Page: 10
Daily proportion of patient-facing hospital HCW infected in 'vaccine rollout' and 'no vaccine' simulation scenarios, by NHSE region.

Supplementary Figure F: Page: 11
Effect of vaccines on infections in susceptible patient-facing hospital HCW, in simulation scenarios, by NHSE region.

Susceptible SIREN participants from the devolved administrations:

For the 578 SIREN participants that met the study criteria relating to susceptibility to primary infection during the second wave, but who were not from England (see Figure 1 of the main manuscript), we calculated the crude attack rates by nation using the same methodology as for Tables 1 and 2 of the main manuscript.

Supplementary Table A: Crude attack rates for susceptible participants from devolved administrations, (with participants from England for comparison).

	**Susceptible to primary infection during second wave**		
**Nation**	**Infected (n)**	**Total (n)**	**% Infected**
Northern Ireland	2	23	8.7
Scotland	29	555	5.2
Wales	No participants enrolled before 8 December 2020		
England	*2353*	*18284*	*12.9*

Susceptible SIREN participants with missing characteristic data:

For the 311 SIREN participants who met the criteria for this study but excluded from the risk factor analysis due to missing or uninformative characteristic data (see Figure 1 of the main manuscript), we calculated the crude attack rates using the same methodology as for Table 1 and 2 of the main manuscript for each of these characteristics. These 311 participants may meet more than one of the missing/uninformative data exclusion criteria.

Supplementary Table B: Crude attack rates for subgroups excluded from the risk factor (regression) analysis due to missing or uninformative characteristic data.

Participant characteristic subgroups excluded from the risk factor analysis		Susceptible to primary infection during second wave		
**Characteristic**	**Subgroup**	**Infected (n)**	**Total (n)**	**% Infected**
Gender	Prefer not to say	1	9	11.1
Ethnicity	Prefer not to say	3	37	8.1
Household size	Prefer not to say	4	49	8.2
Children in household	Prefer not to say	3	32	9.4
IMD quintile (home postcode)	Did not respond	27	219	12.3

Regional comparison of patterns of weekly incidence and vaccination coverage for study participants, and weekly incidence by variant in the general population:

In Figure 2 of the main manuscript we reported the weekly incidence of SARS-CoV-2 primary infections, and weekly cumulative vaccination coverage in SIREN participants susceptible to primary infection during the second wave. Here (Supplementary Figure A) we present the same analyses, but stratified by the same regions used in Figure 3 of the main manuscript. We have also added weekly total population primary incidence (PCR+ or LFD with PCR confirmation) for each region (former Government Offices for the Regions), taken from the UK Health Security Agency (UKHSA) Second Generation Surveillance System (SGSS).

During the second wave, the Alpha variant emerged and was included in genomic surveillance data, with S gene target failure (SGTF) in the Taqpath assay used as a proxy for its identification during this period (1 September 2020 – 30 April 2021)[1]. Therefore the proportion of cases with SGTF, from those specimens analysed in this manner from UK Lighthouse laboratories pillar 2 (community testing), is an estimate for the proportion of Alpha variant infections in the general population.[2]

The SIREN participant and population incidence values in each of the regions are represented as a proportion of their peak weekly values in that region, and for graphical clarity the population figures are represented on a half-scale. We have applied colouring to the weekly population primary incidence values using the SGFT weekly proportions stratified by region, to indicate the timing of the Alpha emergence in each region.

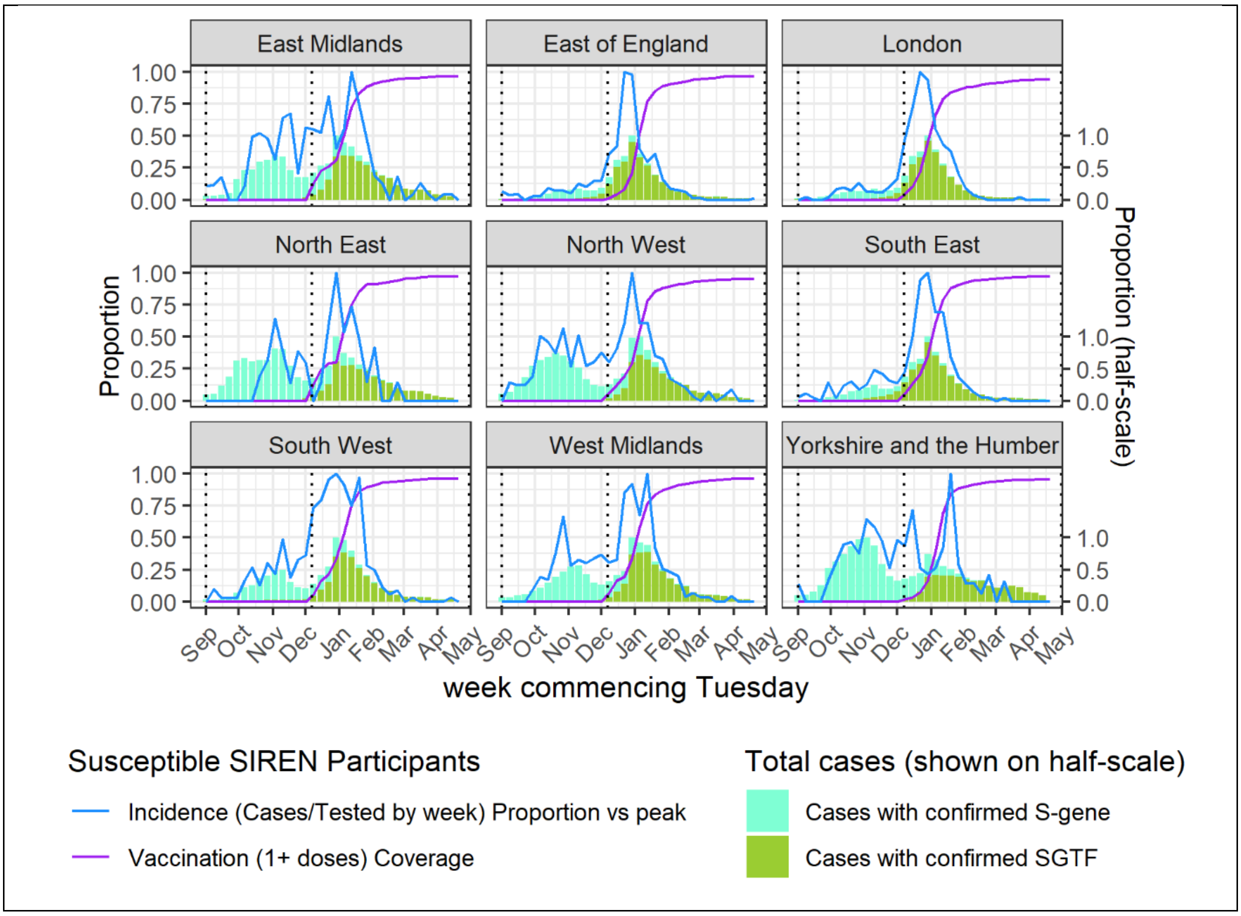

Supplementary Figure A: Weekly incidence and vaccine coverage in susceptible SIREN participants, compared with total incidence stratified by variant, by region. Positive results as a proportion of SIREN participants susceptible to primary infection tested by week (shown as proportion of peak week value) and vaccination coverage (one or more doses) of SIREN participants susceptible to primary infection, weekly count of all COVID-19 cases (PCR+ or LFD with PCR confirmation),[3] with each bar split to indicate an estimate of the proportion of that week's cases attributable to the Alpha variant.

Comparison of patterns of weekly incidence in study participants, in hospital patients, and in the community:

Here (Supplementary Figure B) we place the weekly incidence of primary SARS-CoV-2 infection in susceptible SIREN participants (Figure 2 of the main manuscript) in the context of the overall SARS-CoV-2 incidence in England across the second wave.

We used the methods described in Bhattacharya et al. to distinguish between cases in the community and cases in hospital,[4] with the latter further categorised by an attribution to either hospital-acquired or community-acquired SARS-CoV-2 infection. Bhattacharya's method attributes acquisition (and onset) of cases to hospital or community according to the European Centre for Disease Prevention and Control (ECDC) Surveillance definitions for COVID-19.[5] This is achieved by linking case data (with specimen date), taken from the SGSS, with hospital admission records collated by NHS Digital (from Secondary Uses Service [SUS] and Emergency Care Dataset [ECDS]).

To facilitate comparison of temporal patterns the incidence plots for each category are scaled as a proportion of their peak weekly value.

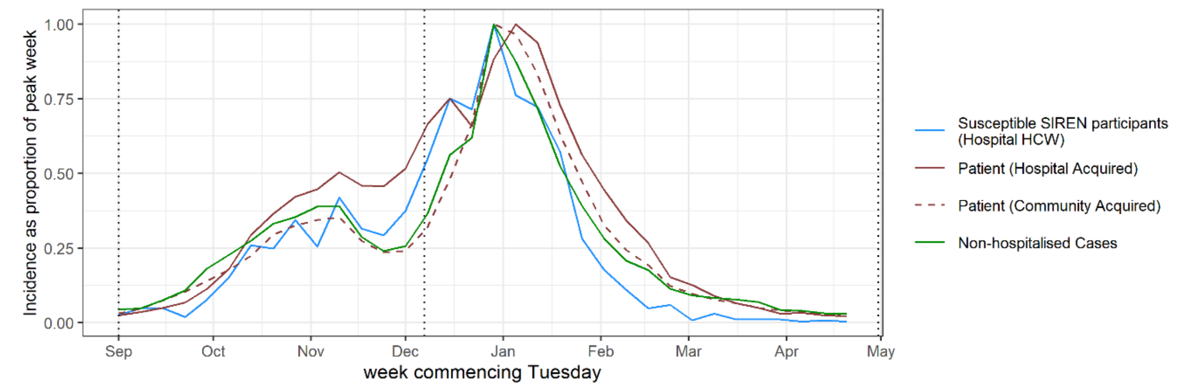

Supplementary Figure B: Weekly incidence in susceptible SIREN participants, hospital patients (acute trusts), and non-hospitalised cases. Allocation of SARS-CoV-2 acquisition uses methodology from Bhattacharya et al.[4] Graphs are scaled so maximum values for each series are plotted at same height.

Mathematical model schematic:

The schematic in Supplementary Figure C1 visually demonstrates a summary of the structure of the mathematical model, as fully described in Appendix 1: Mathematical Modelling Methods.

Two dynamics specific to the second wave were the emergence of the Alpha variant and the start of the vaccine rollout. We therefore present alongside the schematic dynamics, taken from time series data used in the parameterisation of the model, for the proportion of Alpha variant cases and for the proportion of individuals vaccinated.

The parameterisation of the proportion of SARS-CoV-2 cases with the Alpha variant used a logistic curve fitted, by NHSE region, to data published by Volz et al. on the estimated circulation of variants in England,[6] by PCR-tested sample date (Supplementary Figure C2). The COVID-19 vaccination coverage (by number of doses) was parameterised separately for the community and for patient-facing (pfHCW), with the former stratified by age-bands in the Joint Committee on Vaccination and Immunisation (JCVI) prioritisation for primary vaccine in England.[7] The community vaccination coverage parameterisation used daily data, by vaccine date, from National Immunisation Management System (NIMS) for the number of adults in England who received dose(s) of COVID-19 vaccine, with logistics curves fitted to these values as a proportion of the age-band population.[8] The pfHCW vaccination parameterisation used logistic curves fitted to the daily cumulative proportion of SIREN participants who had received dose(s) of COVID-19 vaccine, for each region. Supplementary Figures C3 and C4 show the fitted curves for the cumulative vaccine coverage (one or more doses) by first vaccine date.

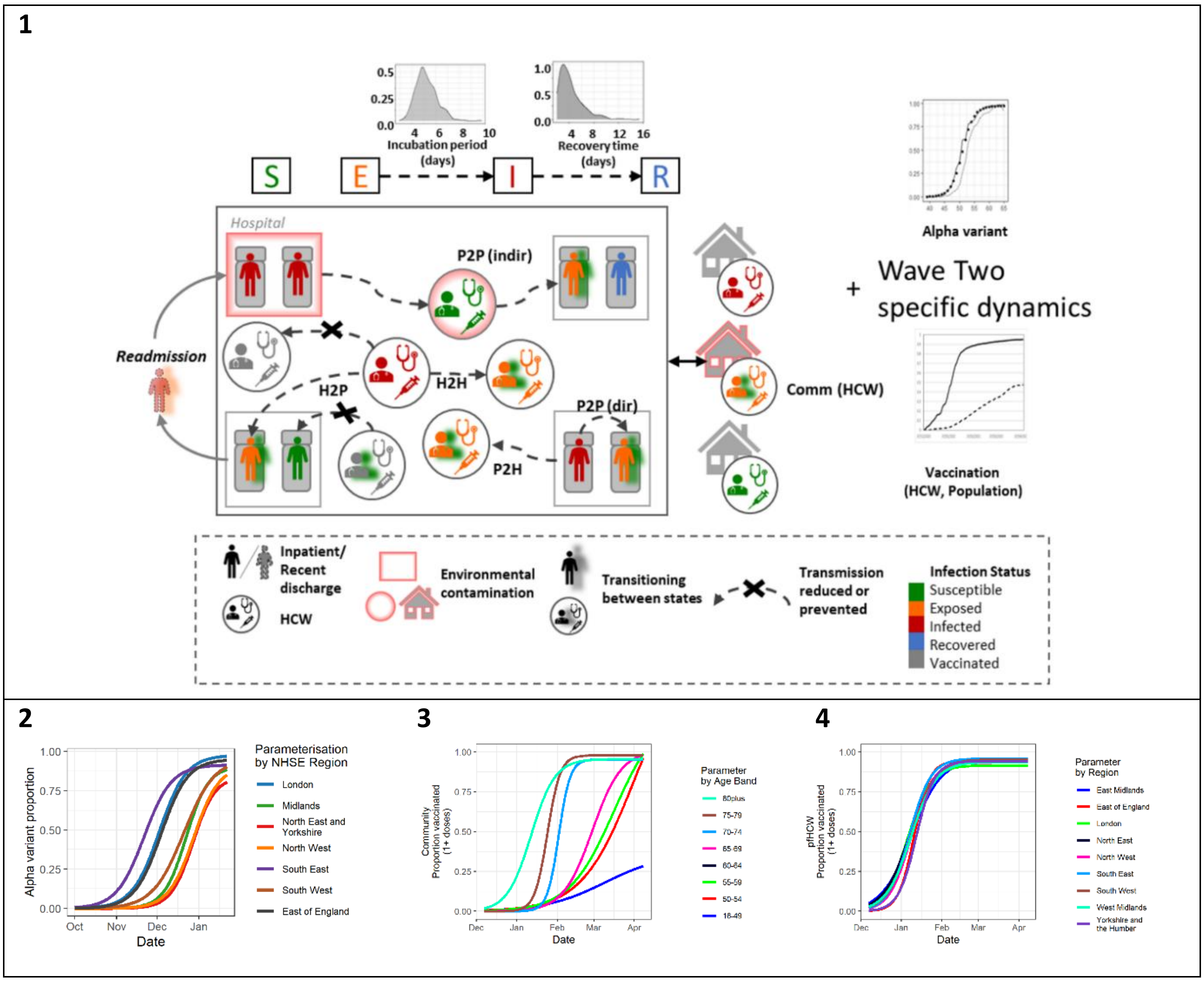

Supplementary Figure C: Mathematical model schematic, and dynamics for daily Alpha variant cases proportion and vaccination coverage. 1) Individuals can be in any of the infection states Susceptible, Exposed (infected but not yet infectious), Infected (infected and infectious, including non-symptomatic), or Recovered, and transition between states probabilistically. Patient-facing hospital HCW can be infected within the hospital by patients or other staff and by the general community while they are not on shift. P2P = patient-to-patient transmission, P2H = patient-to-HCW transmission, H2P = HCW-to-patient transmission, H2H = HCW-to-HCW transmission. 2) Parameter, by NHSE region, for the proportion of new infections that are Alpha variant: logistic curve fitted to data (by PCR sample date) from Volz et al.[6] 3) Parameter, by JVCI prioritisation age-band, for first dose vaccine coverage for individuals in the community: logistic curve fitted to data from NIMS (by first vaccine date). 4) Parameter, by region, for first dose vaccine coverage of pfHCW: logistic curve fitted to data from SIREN/NIMS (by first vaccine date).

Comparing mathematical model simulation output with empirical SIREN participant data:

The mathematical model was calibrated against empirical data for HCW and patients (fully described in Appendix 1: Mathematical modelling methods).

For the HCW calibration, simulation output was calibrated to data on the serological status (ever positive) of SIREN participants study at two timepoints, 10 November 2020 and 8 December 2020 (before the vaccine rollout), with data from 1 September 2020, 12 January 2021, 20 April 2021 being used for validation. The serological status was used as a proxy for cumulative infection – as PCR testing was not routine for asymptomatic cases during the first wave and regular PCR testing for SIREN did not start until June 2020.

At each of these timepoints, the proportion of ever serologically positive participants within each English organisation participating in SIREN was calculated, and median and the distribution of these values used in the calibration process. These distributions are shown in Supplementary Figure D, together with the model output from individual simulation runs giving the simulated cumulative infections in pfHCW.

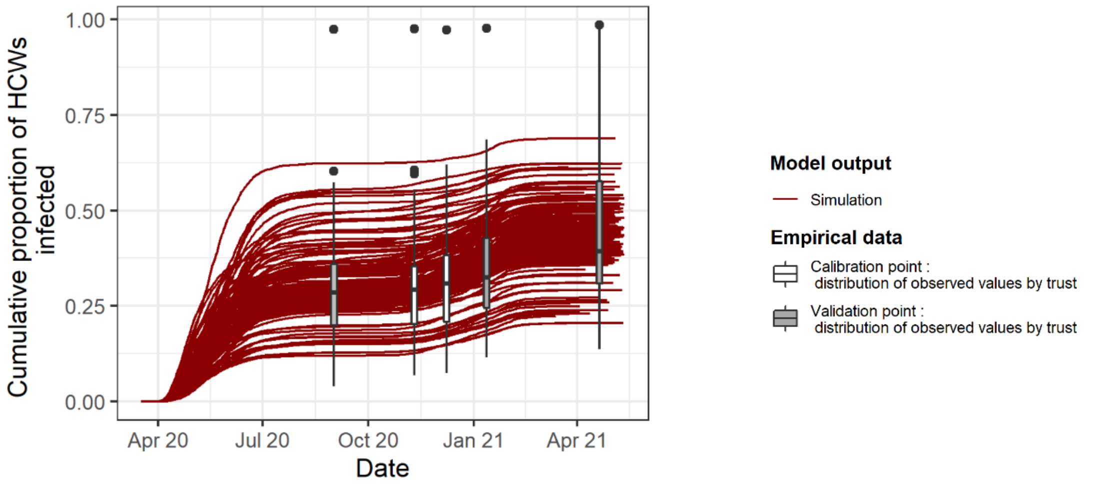

Supplementary Figure D: Cumulative infection in HCW across the pandemic with comparison of mathematical model (IBM) output and SIREN data.

Effect of vaccines on patient-facing hospital HCW (pfHCW) rates of SARS-CoV-2 infection by NHSE region, from simulation output:

In Supplementary Figures E and F we present the same model output as in Figures 5A, 5B and 5D of the main manuscript, but for each NHSE region.

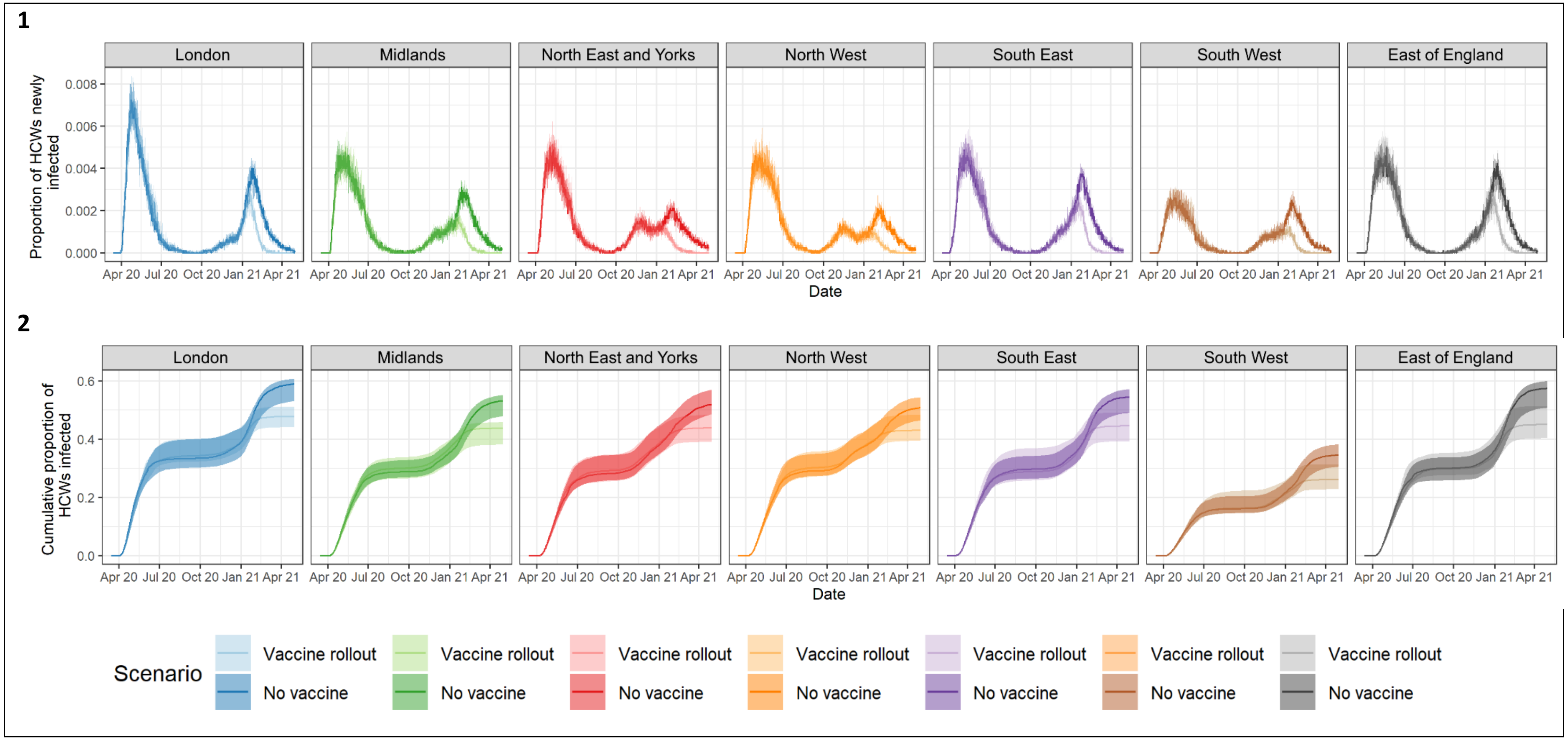

Supplementary Figure E: Daily proportion of patient-facing hospital HCW infected in 'vaccine rollout' and 'no vaccine' simulation scenarios, by NHSE region. 1) Proportion infected across the pandemic, in the 'vaccine rollout' and 'no vaccine' simulation scenarios. 2) Cumulative proportion infected across the pandemic, in the 'vaccine rollout' and 'no vaccine' simulation scenarios.

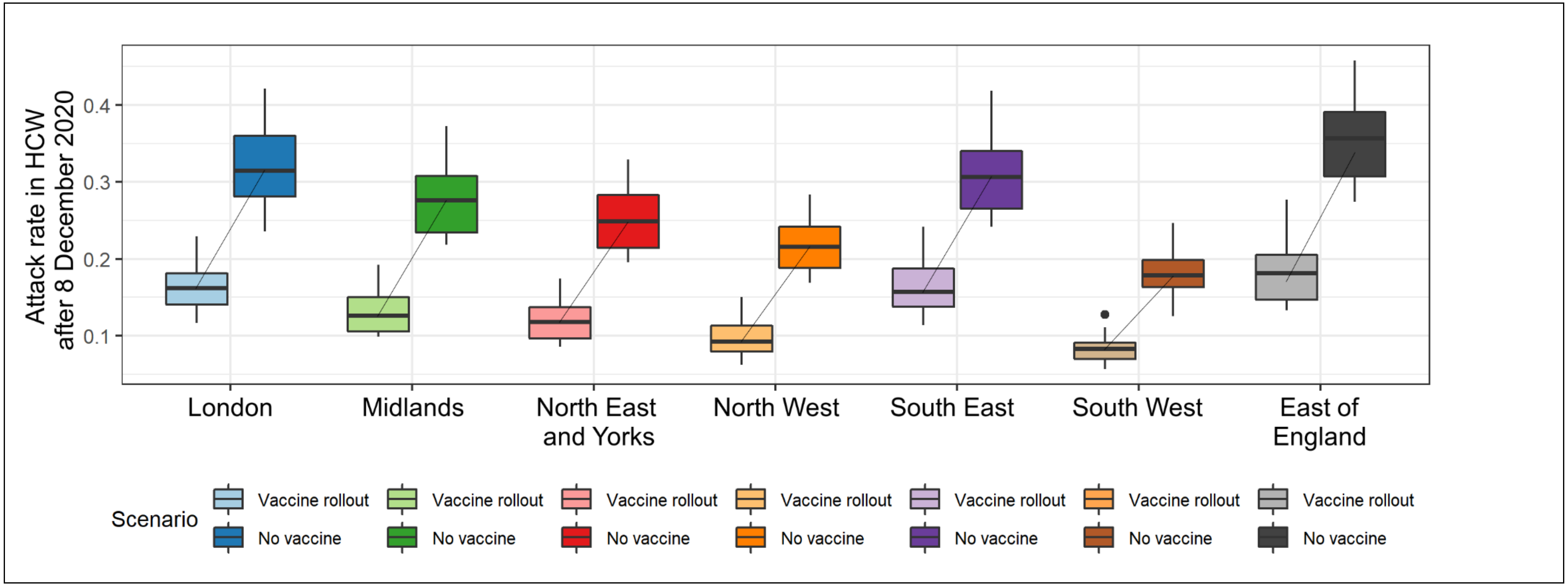

Supplementary Figure F: Effect of vaccines on infections in susceptible patient-facing hospital HCW, in simulation scenarios, by NHSE region. Proportion of susceptible patient-facing hospital HCWs (at 8 December 2020) that were subsequently infected by 30 April 2021 in each simulation scenario. Non-vaccine scenarios are in darker colours and vaccine scenarios are in the lighter colours.

References:

1. Public Health England. Investigation of novel SARS-CoV-2 variant 202012/01: technical briefing 5. https://assets.publishing.service.gov.uk/government/uploads/system/uploads/attachment_data/file/959426/Variant_of_Concern_VOC_202012_01_Technical_Briefing_5.pdf.
2. Public Health England. SARS-CoV-2 variants of concern and variants under investigation in England Technical briefing 9. 22 April 2021. https://assets.publishing.service.gov.uk/government/uploads/system/uploads/attachment_data/file/979818/Variants_of_Concern_VOC_Technical_Briefing_9_England.pdf.
3. UK Health Security Agency. Cases in the UK | Coronavirus in the UK. GOV.UK. https://coronavirus.data.gov.uk/details/cases.
4. Bhattacharya A, Collin SM, Stimson J, et al. Healthcare-associated COVID-19 in England: a national data linkage study. *J Infect* 2021. doi: 10.1016/j.jinf.2021.08.039.
5. European Centre for Disease Prevention and Control. Surveillance definitions for COVID-19. https://www.ecdc.europa.eu/en/covid-19/surveillance/surveillance-definitions.
6. Volz E, Mishra S, Chand M, et al. Assessing transmissibility of SARS-CoV-2 lineage B.1.1.7 in England. *Nature* 2021;593(7858):266-69. doi: 10.1038/s41586-021-03470-x.
7. Joint Committee on Vaccination and Immunisation. Joint Committee on Vaccination and Immunisation: advice on priority groups for COVID-19 vaccination, 30 December 2020. 6 January 2021. https://www.gov.uk/government/publications/priority-groups-for-coronavirus-covid-19-vaccination-advice-from-the-jcvi-30-december-2020/joint-committee-on-vaccination-and-immunisation-advice-on-priority-groups-for-covid-19-vaccination-30-december-2020.
8. Office for National Statistics. National population projections: 2018-based. Office for National Statistics. https://www.ons.gov.uk/peoplepopulationandcommunity/populationandmigration/populationprojections/bulletins/nationalpopulationprojections/2018based.
