# Supplementary material for: Burden of SARS-CoV-2 infection in healthcare workers during second wave in England and impact of vaccines: prospective multicentre cohort study (SIREN) and mathematical model
Source: BMJ. 2022 Jul 20;378:e070379. doi: 10.1136/bmj-2022-070379 (PMC9295077; doi:10.1136/bmj-2022-070379)
Supplement: Supplementary file 3 — Web appendix: Appendix 3: SIREN Study Group members [file popd070379.ww3.pdf]

Appendix 3: SIREN Study Group Members.

SIREN Study Group (UKHSA team and Devolved Administrations), SIREN associated studies and participating SIREN site members:

No.	SIREN Study Group	First name	Surname
1.	UK Health Security Agency	Ana	Atti
2.	UK Health Security Agency	Omoyeni	Adebiyi
3.	UK Health Security Agency	Nick	Andrews
4.	UK Health Security Agency	Tim	Brooks
5.	UK Health Security Agency	Colin	Brown
6.	UK Health Security Agency	Davina	Calbraith
7.	UK Health Security Agency	Meera	Chand
8.	UK Health Security Agency	Andre	Charlett
9.	UK Health Security Agency	Michelle	Cole
10.	UK Health Security Agency	Joanna	Conneely
11.	UK Health Security Agency	Paul	Conneely
12.	UK Health Security Agency	Silvia	D'Arcangelo
13.	UK Health Security Agency	Sarah	Foulkes
14.	UK Health Security Agency	Nabila	Fowles-Gutierrez
15.	UK Health Security Agency	Eileen	Gallagher
16.	UK Health Security Agency	Victoria	Hall
17.	UK Health Security Agency	Nipunadi	Hettiarachchi
18.	UK Health Security Agency	Jacqueline	Hewson
19.	UK Health Security Agency	Susan	Hopkins
20.	UK Health Security Agency	Kate	Howell
21.	UK Health Security Agency	Ferdinando	Insalata
22.	UK Health Security Agency	Jasmin	Islam
23.	UK Health Security Agency	Jameel	Khawam
24.	UK Health Security Agency	Robert	Kyffin
25.	UK Health Security Agency	Ezra	Linley
26.	UK Health Security Agency	Iain	Milligan
27.	UK Health Security Agency	Edward	Monk
28.	UK Health Security Agency	Katie	Munro
29.	UK Health Security Agency	Claire	Neill
30.	UK Health Security Agency	Anne-Marie	O'Connell
31.	UK Health Security Agency	Ashley	Otter
32.	UK Health Security Agency	Mary	Ramsay
33.	UK Health Security Agency	Cathy	Rowe
34.	UK Health Security Agency	Ayoub	Saei
35.	UK Health Security Agency	Noshin	Sajedi
36.	UK Health Security Agency	Amanda	Semper
37.	UK Health Security Agency	Andrew	Taylor-Kerr
38.	UK Health Security Agency	Jean	Timeyin
39.	UK Health Security Agency	Simon	Tonge
40.	UK Health Security Agency	Caio	Tranquillini
41.	UK Health Security Agency	Edgar	Wellington
42.	UK Health Security Agency	Maria	Zambon
43.	Public Health Agency Northern Ireland	Dianne	Corrigan
44.	Public Health Agency Northern Ireland	Lisa	Cromey
45.	Glasgow Caledonian University & Public Health Scotland	Lesley	Price
46.	Public Health Scotland	Josie	Long
47.	Glasgow Caledonian University & Public Health Scotland	Nicola	Sergenson

48.	Public Health Scotland	Jennifer	Bishop
49.	Public Health Scotland	Jennifer	Weir
50.	Glasgow Caledonian University	Ayo	Matuluko
51.	Glasgow Caledonian University	Annelysse	Jorgenson
52.	Public Health Scotland	Laura	Dobbie
53.	Public Health Scotland	Andrew	Telfer
54.	Public Health Scotland	David	Goldberg
55.	Public Health Wales	Ellen	de Lacy
56.	Public Health Wales	Guy	Stevens
57.	Public Health Wales	Susannah	Froude
58.	Public Health Wales	Linda	Tyson
59.	Health and Care Research Wales	Yvette	Ellis
60.	Health and Care Research Wales	Chris	Norman
**No.**	**SIREN Associated Studies**	**First name**	**Surname**
1.	Protective Immunity from T cells to Covid-19 in Health workers (PITCH)	Susanna	Dunachie
2.	Protective Immunity from T cells to Covid-19 in Health workers (PITCH)	Paul	Klenerman
3.	Protective Immunity from T cells to Covid-19 in Health workers (PITCH)	Chris	Duncan
4.	Protective Immunity from T cells to Covid-19 in Health workers (PITCH)	Lance	Turtle
5.	Protective Immunity from T cells to Covid-19 in Health workers (PITCH)	Alex	Richter
6.	Protective Immunity from T cells to Covid-19 in Health workers (PITCH)	Thushan	de Silva
7.	Protective Immunity from T cells to Covid-19 in Health workers (PITCH)	Eleanor	Barnes
8.	Protective Immunity from T cells to Covid-19 in Health workers (PITCH)	Daniel	Wootton
9.	The Humoral Immune Correlates for COVID-19 (HICC) consortium	Jonathan	Heeney
10.	The Humoral Immune Correlates for COVID-19 (HICC) consortium	Helen	Baxendale
11.	The Humoral Immune Correlates for COVID-19 (HICC) consortium	Javier	Castillo-Olivares
12.	The Francis Crick Institute	Rupert	Beale
13.	The Francis Crick Institute	Edward	Carr
14.	Genotype2Phenotype (G2P)	Wendy	Barclay
15.	Genotype2Phenotype (G2P)	Massimo	Palmarini
16.	GenOMICC	John Kenneth	Baillie
**No.**	**Participating SIREN Sites**	**First name**	**Surname**
1.	ALDER HEY CHILDREN'S NHS FOUNDATION TRUST	B.	Larru
		S.	Mcwilliam
2.	ANEURIN BEVAN UNIVERSITY LHB	Anna	Roynon
		Maxine	Nash
3.	ASHFORD AND ST PETER'S HOSPITALS NHS FOUNDATION TRUST	Stephen	Winchester
		Talat	Akhtar
4.	BASILDON AND THURROCK UNIVERSITY HOSPITALS NHS FOUNDATION TRUST	Stacey	Pepper
		Georgina	Butt
5.	BEDFORDSHIRE HOSPITALS NHS FOUNDATION TRUST	Simantee	Guha
		Philippa	Bakker
6.	BELFAST HEALTH AND SOCIAL CARE TRUST	Clodagh	Loughrey
		A.	Watt

7.	BETSI CADWALADR UNIVERSITY LHB	Julia	Roberts
		Caroline	Mulvaney Jones
8.	BIRMINGHAM AND SOLIHULL MENTAL HEALTH NHS FOUNDATION TRUST	Manny	Bagary
		Siobhan	Keogh
9.	BIRMINGHAM COMMUNITY HEALTHCARE NHS FOUNDATION TRUST	Claire	Williams
		April	Hawkins
10.	BLACK COUNTRY HEALTHCARE NHS FOUNDATION TRUST	Alison	Grant
		Rebecca	Temple-Purcell
11.	BLACKPOOL TEACHING HOSPITALS NHS FOUNDATION TRUST	Joanne	Howard
		Emma	Ward
12.	BOLTON NHS FOUNDATION TRUST	Scott	Latham
		Raksha	Mistry
13.	BRIGHTON AND SUSSEX UNIVERSITY HOSPITALS NHS TRUST	Lisa	Barbour
		Helena	Sovriarova
14.	BUCKINGHAMSHIRE HEALTHCARE NHS TRUST	R.	Penn
		N.	Wong
15.	CALDERDALE AND HUDDERSFIELD NHS FOUNDATION TRUST	G.	Boyd
		A.	Rajgopal
16.	CENTRAL AND NORTH WEST LONDON NHS FOUNDATION TRUST	Alejandro	Arenas-Pinto
		Abigail	Severn
17.	CHESTERFIELD ROYAL HOSPITAL NHS FOUNDATION TRUST	Edward	Harris
		Amanda	Whileman
18.	CORNWALL PARTNERSHIP NHS FOUNDATION TRUST	Richard	Laugharne
		Susan	Greenwood
19.	COUNTESS OF CHESTER HOSPITAL NHS FOUNDATION TRUST	T.	Barnes
		C.	Jones
20.	CROYDON HEALTH SERVICES NHS TRUST	Banerjee	SubhroOsuji
		Anna	Rokakis
21.	CWM TAF MORGANNWG UNIVERSITY LHB	John	Geen
		Carla	Pothecary
22.	DARTFORD AND GRAVESHAM NHS TRUST	Tracy	Edmunds
		Nihil	Chitalia
23.	DERBYSHIRE COMMUNITY HEALTH SERVICES NHS FOUNDATION TRUST	Kim	Gray
		Eve	Etell Kirby
24.	DERBYSHIRE HEALTHCARE NHS FOUNDATION TRUST	S.	Akhtar
		G.	Harrison
25.	DEVON PARTNERSHIP NHS TRUST	Clare	McAdam
		Stacey	Horne
26.	DONCASTER AND BASSETLAW TEACHING HOSPITALS NHS FOUNDATION TRUST	K.	Agwuh
		V.	Maxwell
27.	DORSET COUNTY HOSPITAL NHS FOUNDATION TRUST	Jennifer	Graves
28.	DORSET HEALTHCARE UNIVERSITY NHS FOUNDATION TRUST	James	Colton
		Stephanie	Willshaw
29.	EAST SUFFOLK AND NORTH ESSEX NHS FOUNDATION TRUST	A.	O'Kelly
		P.	Ridley
30.	EAST SUSSEX HEALTHCARE NHS TRUST	Anna	Cowley
		Janet	Sinclair
31.	EPSOM AND ST HELIER UNIVERSITY HOSPITALS NHS TRUST	Helen	Johnstone
		Neringa	Vilimiene
32.	FRIMLEY HEALTH NHS FOUNDATION TRUST	Manjula	Meda
		Jane	Democratis
33.	GEORGE ELIOT HOSPITAL NHS TRUST	Simon	Brake
		David	Boss
34.	GLOUCESTERSHIRE HOSPITALS NHS FOUNDATION TRUST	Christine	Ford
		Steve	Hams
35.	GOLDEN JUBILEE NATIONAL HOSPITAL	Catherine	Sinclair
		Val	Irvine

36.	GREAT WESTERN HOSPITALS NHS FOUNDATION TRUST	Eva	Fraile
		Badrinathan	Chandrasekaran
37.	HAMPSHIRE HOSPITALS NHS FOUNDATION TRUST	Claire	Thomas
		Ina	Hoad
38.	HOUNSLOW AND RICHMOND COMMUNITY HEALTHCARE NHS TRUST	Shekoo	Mackay
		Shivani	Khan
39.	HULL UNIVERSITY TEACHING HOSPITALS NHS TRUST	Philippa	Burns
		Nicholas	Easom
40.	HYWEL DDA UNIVERSITY LHB	Tracy	Lewis
		Zohra	Omar
41.	IMPERIAL COLLEGE HEALTHCARE NHS TRUST	Graham	Pickard
		Kenisha	Lewis
42.	ISLE OF WIGHT NHS TRUST	Sarah	Hinch
		Alison	Brown
43.	JAMES PAGET UNIVERSITY HOSPITALS NHS FOUNDATION TRUST	Ben	Burton
		Christian	Hacon
44.	KING'S COLLEGE HOSPITAL NHS FOUNDATION TRUST	Ray	Chaudhuri
		Jonnie	Aeron-Thomas
45.	LANCASHIRE & SOUTH CUMBRIA NHS FOUNDATION TRUST	Robert	Shorten
		Kathryn	Hollinshead
46.	LANCASHIRE TEACHING HOSPITALS NHS FOUNDATION TRUST	Claire	Corless
		Robert	Shorten
47.	LEEDS TEACHING HOSPITALS NHS TRUST	Kyra	Holliday
		Clair	Favager
48.	LEICESTERSHIRE PARTNERSHIP NHS TRUST	Sarah	Baillon
		Joanne	Edgar
49.	LEWISHAM AND GREENWICH NHS TRUST	A.	Shah
		J.	Russell
50.	LINCOLNSHIRE PARTNERSHIP NHS FOUNDATION TRUST	Kelly	Moran
		Ananta	Dave
51.	LIVERPOOL UNIVERSITY HOSPITALS NHS FOUNDATION TRUST	Anu	Chawla
		Fran	Westwell
52.	LONDON NORTH WEST UNIVERSITY HEALTHCARE NHS TRUST	Ekaterina	Watson
		David	Adeboyeku
53.	MAIDSTONE AND TUNBRIDGE WELLS NHS TRUST	C.	Pegg
		M.	Williams
54.	MANCHESTER UNIVERSITY NHS FOUNDATION TRUST	S.	Ahmad
		A.	Horsley
55.	MID CHESHIRE HOSPITALS NHS FOUNDATION TRUST	Murray	Luckas
		Diego	Maseda
56.	MID ESSEX HOSPITAL SERVICES NHS TRUST	Lauren	Sach
		Yvonne	Lester
57.	MID YORKSHIRE HOSPITALS NHS TRUST	Ismaelette	Del Rosario
		John	Ashcroft
58.	MOORFIELDS EYE HOSPITAL NHS FOUNDATION TRUST	Roxanne	Crosby-Nwaobi
		Chloe	Reeks
59.	NHS BORDERS	Joy	Dawson
		Lauren	Finlayson
60.	NHS FIFE	Susan	Fowler
		Devesh	Dhasmana
61.	NHS FORTH VALLEY	Euan	Cameron
		Anne	Todd
62.	NHS GRAMPIAN	Lynne	Walker
		Vhairi	Bateman
63.	NHS GREATER GLASGOW AND CLYDE	Antonia	Ho
		Michael	Murphy
64.	NHS HIGHLAND	David	Dytmer
		Alexandra	Cochrane

65.	NHS LANARKSHIRE	Manish	Patel
		Karen	Black
66.	NHS LOTHIAN	Kate	Templeton
		Jane	Crowe
67.	NHS WESTERN ISLES	Martin	Malcolm
		Joan	Frieslick
68.	NORFOLK AND NORWICH UNIVERSITY HOSPITALS NHS FOUNDATION TRUST	Ngozi	Elumogo
		Louise	Coke
69.	NORTH CUMBRIA INTEGRATED CARE NHS FOUNDATION TRUST	Beverly	Wilkinson
		John	Elliott
70.	NORTH MIDDLESEX UNIVERSITY HOSPITAL NHS TRUST	Mariyam	Mirfenderesky
		Pratap	Harbham
71.	NORTH WEST ANGLIA NHS FOUNDATION TRUST	Janki	Bhayani
		Stephanie	Diaz
72.	NORTHERN DEVON HEALTHCARE NHS TRUST	M.	Howard
		T.	Lewis
73.	NORTHERN HEALTH AND SOCIAL CARE TRUST	Elinor	Hanna
		Frances	Johnston
74.	NORTHERN LINCOLNSHIRE AND GOOLE NHS FOUNDATION TRUST	Jonathan	Hatton
		Peter	Cowling
75.	NOTTINGHAM UNIVERSITY HOSPITALS NHS TRUST	Sarah	Brand
		Imogen	Gould
76.	POOLE HOSPITAL NHS FOUNDATION TRUST	Megan	Woolcook
		Maxine	Ashton
77.	PORTSMOUTH HOSPITALS NHS TRUST	Allison	Dimmer
		Karen	Hudson
78.	POWYS TEACHING LHB	Jayne	Goodwin
		Chris	Norman
79.	QUEEN VICTORIA HOSPITAL NHS FOUNDATION TRUST	J.	Giles
		G.	Pottinger
80.	ROYAL BERKSHIRE NHS FOUNDATION TRUST	Maya	Joseph
		Holly	Coles
81.	ROYAL CORNWALL HOSPITALS NHS TRUST	H.	Chenoweth
		D.	Browne
82.	ROYAL DEVON AND EXETER NHS FOUNDATION TRUST	Cressida	Auckland
		Stephanie	Prince
83.	ROYAL FREE LONDON NHS FOUNDATION TRUST	Alison	Rodger
		Tabitha	Mahungu
84.	ROYAL NATIONAL ORTHOPAEDIC HOSPITAL NHS TRUST	Esther	Hanison
		Simon	Warren
85.	ROYAL PAPWORTH HOSPITAL NHS FOUNDATION TRUST	Allison	Doel
		Kitty	Paques
86.	ROYAL SURREY COUNTY HOSPITAL NHS FOUNDATION TRUST	Charlie	Piercy
		Esther	Tarr
87.	ROYAL UNITED HOSPITALS BATH NHS FOUNDATION TRUST	Julia	Vasant
		Deborah	Howcroft
88.	SALISBURY NHS FOUNDATION TRUST	Catherine	Thompson
		Sophia	Strong-Sheldrake
89.	SANDWELL AND WEST BIRMINGHAM HOSPITALS NHS TRUST	Masood	Aga
		James	Pethick
90.	SHEFFIELD CHILDREN'S NHS FOUNDATION TRUST	S.	Gormley
		C.	Kerrison
91.	SHEFFIELD TEACHING HOSPITALS NHS FOUNDATION TRUST	Thushan	de Silva
		Simon	Tazzyman
92.	SHERWOOD FOREST HOSPITALS NHS FOUNDATION TRUST	Lynne	Allsop
		Shrikant	Ambalkar
93.	SHREWSBURY AND TELFORD HOSPITAL NHS TRUST	Mandy	Beekes
		Hannah	Gibson

94.	SHROPSHIRE COMMUNITY HEALTH NHS TRUST	Johanne	Tomlinson
95.	SOLENT NHS TRUST	Cathy	Price
		The Solent Research Team	
96.	SOMERSET NHS FOUNDATION TRUST	Justin	Pepperell
		Kate	James
97.	SOUTH EASTERN HEALTH AND SOCIAL CARE	Yuri	Protaschik
		Tom	Trinick
98.	SOUTHEND UNIVERSITY HOSPITAL NHS FOUNDATION TRUST	Paula	Harman
		Joanne	Galliford
99.	SOUTHERN HEALTH AND SOCIAL CARE TRUST	Angel	Boulos
		Fiona	Thompson
100.	SOUTHERN HEALTH NHS FOUNDATION TRUST	Qi	Zheng
		Danielle	McCracken
101.	SOUTHPORT AND ORMSKIRK HOSPITAL NHS TRUST	Katherine	Gray
		Kerryanne	Brown
102.	ST GEORGE'S UNIVERSITY HOSPITALS NHS FOUNDATION TRUST	Tim	Planche
		Angela	Houston
103.	ST HELENS AND KNOWSLEY TEACHING HOSPITALS NHS TRUST	Rowan	Pritchard Jones
		Diane	Wycherley
104.	STOCKPORT NHS FOUNDATION TRUST	Barzo	Faris
105.	SURREY AND SUSSEX HEALTHCARE NHS TRUST	K.	Nimako
		B.	Stewart
106.	SWANSEA BAY UNIVERSITY LHB	Claire	Stafford
		Steve	Bain
107.	THE CLATTERBRIDGE CANCER CENTRE NHS FOUNDATION TRUST	Sheena	Khanduri
		Nagesh	Kalakonda
108.	THE DUDLEY GROUP NHS FOUNDATION TRUST	Helen	Ashby
109.	THE HILLINGDON HOSPITALS NHS FOUNDATION TRUST	Natasha	Mahabir
110.	THE NEWCASTLE UPON TYNE HOSPITALS NHS FOUNDATION TRUST	J.	Harwood
		B.	Payne
111.	THE PRINCESS ALEXANDRA HOSPITAL NHS TRUST	Kathryn	Court
		Nikki	White
112.	THE ROBERT JONES AND AGNES HUNT ORTHOPAEDIC HOSPITAL NHS FOUNDATION TRUST	Ruth	Longfellow
113.	THE ROYAL BOURNEMOUTH AND CHRISTCHURCH HOSPITALS NHS FOUNDATION TRUST	Mihye	Lee
114.	THE ROYAL WOLVERHAMPTON NHS TRUST	Marie	Green
		Lauren	Hughes
115.	TORBAY AND SOUTH DEVON NHS FOUNDATION TRUST	Mathew	Halkes
		Pauline	Mercer
116.	UNITED LINCOLNSHIRE HOSPITALS NHS TRUST	Alun	Roebuck
		ULHT Research Team	
117.	UNIVERSITY HOSPITAL SOUTHAMPTON NHS FOUNDATION TRUST	E.	Wilson-Davies
118.	UNIVERSITY HOSPITALS BRISTOL AND WESTON NHS FOUNDATION TRUST	Rajeka	Lazarus
		Aaran	Sinclair
119.	UNIVERSITY HOSPITALS COVENTRY AND WARWICKSHIRE NHS TRUST	N.	Aldridge
		L.	Berry
120.	UNIVERSITY HOSPITALS OF DERBY AND BURTON NHS FOUNDATION TRUST	F.	Game
		T.	Reynolds
121.	UNIVERSITY HOSPITALS OF LEICESTER	Christopher	Holmes

	NHS TRUST	Martin	Wiselka
122.	UNIVERSITY HOSPITALS OF MORECAMBE BAY NHS FOUNDATION TRUST	Andrew	Higham
		Lisa	Bishop
123.	UNIVERSITY HOSPITALS OF NORTH MIDLANDS NHS TRUST	Christopher	Duff
		Joanne	Gray
124.	UNIVERSITY HOSPITALS PLYMOUTH NHS TRUST	Hannah	Jory
		Penny	Harris
125.	VELINDRE NHS Trust	Charlotte	Young
		James	Powell
126.	WALSALL HEALTHCARE NHS TRUST	Lisa	Richardson
		Aiden	Plant
127.	WARRINGTON AND HALTON TEACHING HOSPITALS NHS FOUNDATION TRUST	Zaman	Qazzafi
		Lisa	Ditchfield
128.	WEST SUFFOLK NHS FOUNDATION TRUST	A.	Moody
		R.	Tilley
129.	WESTERN HEALTH AND SOCIAL CARE TRUST	Tracy	Donaghy
		Maurice	O'Kane
130.	WESTERN SUSSEX HOSPITALS NHS FOUNDATION TRUST	R.	Sierra
		K.	Shipman
131.	WHITTINGTON HEALTH NHS TRUST	Philippa	Kemsley
		Zehra’a	Al-Khafaji
132.	WIRRAL UNIVERSITY TEACHING HOSPITAL NHS FOUNDATION TRUST	D.	Harvey
		Y.	Huang
133.	WYE VALLEY NHS TRUST	L.	Robinson
134.	YEOVIL DISTRICT HOSPITAL NHS FOUNDATION TRUST	Sarah	Board
		Andrew	Broadley
135.	YORK TEACHING HOSPITAL NHS FOUNDATION TRUST	Claire	Brookes
		Mags	Szewczyk
